# Protein Disulphide Isomerase A1 Is Involved in the Regulation of Breast Cancer Cell Adhesion and Transmigration via Lung Microvascular Endothelial Cells

**DOI:** 10.3390/cancers12102850

**Published:** 2020-10-02

**Authors:** Marta Stojak, Magdalena Milczarek, Anna Kurpinska, Joanna Suraj-Prazmowska, Patrycja Kaczara, Kamila Wojnar-Lason, Joanna Banach, Martyna Stachowicz-Suhs, Joanna Rossowska, Ivars Kalviņš, Joanna Wietrzyk, Stefan Chlopicki

**Affiliations:** 1 Jagiellonian Centre for Experimental Therapeutics (JCET), Jagiellonian University, 30-348 Krakow, Poland; marta.stojak@jcet.eu (M.S.); anna.kurpinska@jcet.eu (A.K.); joanna.suraj@jcet.eu (J.S.-P.); patrycja.kaczara@jcet.eu (P.K.); kamila.wojnar-lason@jcet.eu (K.W.-L.); 2 Department of Experimental Oncology, Hirszfeld Institute of Immunology and Experimental Therapy, Polish Academy of Sciences, 53-114 Wroclaw, Poland; magdalena.milczarek@hirszfeld.pl (M.M.); joanna.banach@hirszfeld.pl (J.B.); martyna.stachowicz@hirszfeld.pl (M.S.-S.); joanna.rossowska@hirszfeld.pl (J.R.); 3 Department of Pharmacology, Jagiellonian University Medical College, 31-531 Krakow, Poland; 4 Laboratory of Carbofunctional Compounds, Latvian Institute of Organic Synthesis, LV-1006 Riga, Latvia; kalvins@osi.lv

**Keywords:** protein disulphide isomerase A1, adhesion, transendothelial migration, disulphide exchange

## Abstract

**Simple Summary:**

Metastasis is one of the most devastating aspect of cancer progression and involves biochemical and physical interactions between cancer cells and surrounding microenvironment. In particular, cancer cells adhesion to endothelium and their subsequent transendothelial migration represent important steps in the metastatic process in target organs. In this study we characterized the functional role of Protein Disulfide Isomerase A1 (PDIA1) in breast cancer cells adhesion and transendothelial migration. We identified the full repertoire of protein disulfide isomerases in endothelial cells as well as in breast cancer cells. We provided insight into the mechanisms involved in cancer-endothelial cells interactions and suggested that PDIA1 regulates the adhesion and transendothelial migration of breast cancer cells by disulphide exchange involving most likely the activation of integrins. Our results suggest that the inhibition of extracellular PDIA1 or other PDIs represents an interesting target for anti-metastatic treatment.

**Abstract:**

Cancer cell cross-talk with the host endothelium plays a crucial role in metastasis, but the underlying mechanisms are still not fully understood. We studied the involvement of protein disulphide isomerase A1 (PDIA1) in human breast cancer cell (MCF-7 and MDA-MB-231) adhesion and transendothelial migration. For comparison, the role of PDIA1 in proliferation, migration, cell cycle and apoptosis was also assessed. Pharmacological inhibitor, bepristat 2a and PDIA1 silencing were used to inhibit PDIA1. Inhibition of PDIA1 by bepristat 2a markedly decreased the adhesion of breast cancer cells to collagen type I, fibronectin and human lung microvascular endothelial cells. Transendothelial migration of breast cancer cells across the endothelial monolayer was also inhibited by bepristat 2a, an effect not associated with changes in ICAM-1 expression or changes in cellular bioenergetics. The silencing of PDIA1 produced less pronounced anti-adhesive effects. However, inhibiting extracellular free thiols by non-penetrating blocker p-chloromercuribenzene sulphonate substantially inhibited adhesion. Using a proteomic approach, we identified that β1 and α2 integrins were the most abundant among all integrins in breast cancer cells as well as in lung microvascular endothelial cells, suggesting that integrins could represent a target for PDIA1. In conclusion, extracellular PDIA1 plays a major role in regulating the adhesion of cancer cells and their transendothelial migration, in addition to regulating cell cycle and caspase 3/7 activation by intracellular PDIA1. PDIA1-dependent regulation of cancer–endothelial cell interactions involves disulphide exchange and most likely integrin activation but is not mediated by the regulation of ICAM-1 expression or changes in cellular bioenergetics in breast cancer or endothelial cells.

## 1. Introduction

Cancer cell metastasis, the major cause of cancer patient death [1], is a complex phenomenon. It involves cancer cell invasion at the primary site, survival and arrest in the bloodstream, and finally, extravasation at a distant site. Cancer cell adhesion and the subsequent transmigration across endothelium to distant organs is a crucial step of metastasis. Organ-specific colonisation is a key feature of most metastatic cancer cells, and breast cancer cells tend to infiltrate the bone, brain, liver and lungs [2,3,4,5]. Despite the extensive literature, the mechanisms that promote the adhesion of breast cancer cells to organ-specific endothelial layers remain unclear. Better understanding of the specific molecular mechanisms used by cancer cells to activate dynamic interactions with endothelial cells is instrumental to identify promising novel molecular targets for anti-metastatic therapy.

Interestingly, protein disulphide isomerases (PDIs), a group of enzymes exhibiting oxidoreductase activity, have been recently demonstrated to be involved in the modulation of adhesive inter-cellular interactions with endothelium [6,7]. Cell-surface or secreted PDIs regulate platelet adhesion and thrombosis [8,9,10] as well as leukocyte adhesion to endothelium [7]. PDIs are now known to reside not only in the endoplasmic reticulum (ER), regulating protein folding by forming, breaking and rearranging disulphide bonds [11]; various PDIs have been detected in other cellular locations, including the surface of platelets, leukocytes, endothelial cells and cancer cells, and they regulate various protein functions by modifying extracellular disulphide bonds [6,11,12,13,14,15,16].

Recent studies have shown that several PDIs, such as PDIA1, PDIA3 and PDIA6, are upregulated in different cancer types, including kidney, lung, brain, ovarian, melanoma, prostate and male germ-cell tumours, and over-expression of PDIs may serve as a diagnostic marker for cancer [11,15,17,18]. In particular, prolyl 4-hydroxylase, β polypeptide (P4HB), also known as PDIA1, is upregulated in numerous types of cancer [19,20,21,22,23], and its over-expression is associated with advanced-stage tumours and poor prognosis. These findings have been explained by showing that PDIA1 was involved in regulating cancer cell malignancy [24], apoptosis [25] and proliferation [10]. However, it is not known whether PDIA1 regulates cancer cell interactions with endothelium, such as adhesion and transmigration, key processes involved in cancer metastasis.

In the present study, we aimed to fill this gap and identify the relative importance of PDIA1 in regulating interactions of human breast cancer cells (MCF-7 and MDA-MB-231) with lung microvascular endothelial cells. For comparison, we evaluated the role of PDIA1 in proliferation, migration and regulation of the cell cycle, apoptosis and cancer growth in colony formation assays. We identified PDIA1 as a major isoform of PDIs present in human breast cancer cells and lung microvascular endothelial cells and studied the effects of PDIA1 inhibitor, bepristat 2a and PDIA1 silencing on cancer–endothelium interactions and other functional aspects of the cancer cell phenotype. We also explored whether the effects of PDIA1 inhibition on cancer–endothelial cell interactions could be attributed to disulphide re-arrangement of integrins known to mediate adhesion of platelets and leukocytes to endothelium [7,26,27,28], to cellular bioenergetics in breast cancer or endothelial cells, an important target for anti-adhesive mechanisms [29], or to alterations in ICAM-1 expression involved in this interaction [30].

## 2. Results

### 2.1. Relative Content of PDIA1 in Comparison to Other Isoforms of PDIs in MDA-MB-231, MCF-7 and hLMVEC Cell Lines

As shown in Table 1, PDIA1 was the most abundant isoform of all PDIs in all three cell types. PDIA3, PDIA4 and PDIA6 content was also relatively high, but lower compared with PDIA1. Other PDI isoforms were expressed at much lower levels. Interestingly, PDIA17 was present in MCF-7 cells at relatively high levels, while in MDA-MB-231, the expression of PDIA17 was low and not detectable in hLMVECs.

### 2.2. Silencing of PDIA1 in MDA-MB-231 and MCF-7 Cell Lines

To inhibit the function of PDIA1 in breast cancer cell lines, sublines with silenced PDIA1 were constructed. The efficacy of the silencing procedure was confirmed by real-time PCR and Western blot. Based on these results, two sublines were selected for further studies (Figure S1, uncropped western blot figures are shown in Figure S8). The silencing of PDIA1 did not affect MDA-MB-231 cell proliferation but significantly decreased MCF-7/shPDIA1-1 cell proliferation compared to shN (Figure S1). To further confirm PDI content after PDIA1 silencing in selected two sublines showing >70% inhibition by real-time PCR and Western blot, proteomic analysis of these transduced cell lines was performed. Table 2 shows that the silencing of PDIA1 in MCF-7 cells was more pronounced using shPDIA1-1 hairpin (>95% downregulation), while shPDIA1-3 had a weaker effect but also proved effective (ca 80% downregulation). In MDA-MB-231, effective silencing of PDIA1 was achieved using shPDIA1-3 hairpin only (ca 80% downregulation), but a reduction in PDIA1 was also noted in the shN group, and in MDA-MB-231/shPDIA1-1, subline expression of PDIA1 was upregulated compared to shN. Importantly, in PDIA1-silenced cells, other PDIs were neither upregulated nor downregulated.

### 2.3. Inhibition of PDIA1 by Bepristat 2a 

To show that bepristat 2a selectively targets PDIA1, insulin reduction assay was used. As shown in Table 3, bepristat 2a selectively inhibited the reductive activity of PDIA1. Bepristat 2a was characterized by much lower IC_50_ for PDIA1 as compared with PDIA3 (2.1 µM for PDIA1, 127 µM for PDIA3). In addition, bepristat 2a did not affect the reductive activity of other PDI isoforms tested, such as PDIA4, PDIA6 and PDIA17. 

### 2.4. Effect of PDIA1 Inhibition on Cell Proliferation

To determine the cellular response to PDIA1 inhibition, several functional assays evaluated cancer cell phenotype. First, to ensure that the observed effects of bepristat 2a on cancer cell adhesion and transendothelial migration were not related to the cytotoxic activity of this compound, a cell viability assay was performed. As illustrated in Figure S2, bepristat 2a applied at concentrations 1–50 µM for 24 h did not affect MCF-7 or MDA-MB-231 cell viability. A decrease in the viability of all tested cell lines was observed after 100 µM of bepristat 2a. However, bepristat 2a applied at a concentration of 50 µM for 48 h influenced MCF-7 and MDA-MB-231 cell viability. To exclude the observed effects of bepristat 2a on cancer cell adhesion, migration and transendothelial migration, being related to the cytotoxic activity of this compound, further experiments were performed using bepristat 2a at a concentration range of 1–50 µM. Next, the effect of PDIA1 inhibition on caspase 3/7 activity in breast cancer cells was explored. In neither MCF-7 nor MDA-MB-231 cell lines did PDIA1 silencing affect the activity of caspase 3/7 (Figure S3). However, in both cell lines, bepristat 2a used at a concentration of 50 µM significantly activated caspase 3/7 (increase of over 20× in MCF-7 and 10× in MDA-MB-231 compared to untreated control after 48 h and similarly after 24 h of incubation). A statistically significant increase in caspase 3/7 activity was also observed at a concentration of 30 µM of bepristat 2a (over 3× increased activity in both MCF-7 and MDA-MB-231 compared to untreated control after 24 and 48 h incubation; Supplementary Figure S3). Finally, the cell cycle distribution of breast cancer cells after PDIA1 inhibition was examined. The most visible changes in the cell cycle of MCF-7 and MDA-MB-231 cells were observed in S phase, 24 and 48 h after bepristat 2a application (Figure S4). These changes were accompanied by reciprocal changes in the sub-G^1^, G_0_/G_1_ and G_2_/M phases, which became more apparent after 48 than 24 h. We also analysed the cell cycle of transduced MCF-7 and MDA-MB-231 cells. The cell cycle of MCF-7 and MDA-MB-231 with silenced PDIA1 was less disrupted compared to the action of bepristat 2a. Similarly to the effects of bepristat 2a, the silencing of PDIA1 resulted in a lower percentage of MCF-7/shPDIA1-1 cells (where the inhibition of PDIA1 was greater than 95%) in the G_2_/M phase, while the changes in the sub-G_1_, G_0_/G_1_ and S phases were less marked. After the silencing of PDIA1 in MDA-MB-231 cells, we found no changes in the G_0_/G_1_, S or G_2_/M phases (Figure S5).

### 2.5. Effect of PDIA1 Inhibition on Clonogenic Capacity of Breast Cancer Cells

**Figure 1:**
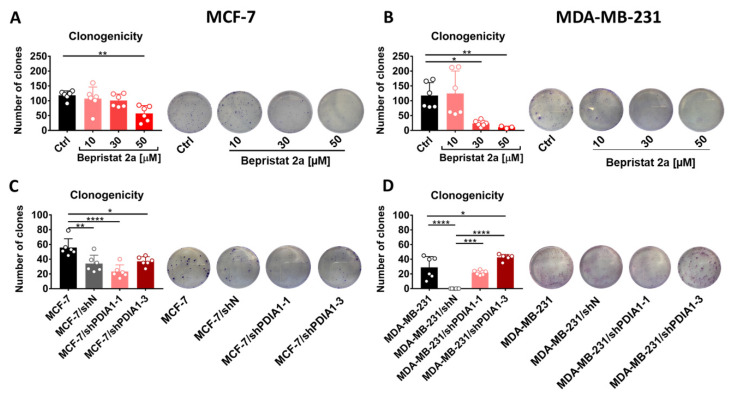
Effect of PDIA1 inhibition on clonogenic capacity of breast cancer cells. The clonogenicity of MCF-7 (**A**) and MDA-MB-231 (**B**) cells treated with bepristat 2a as well as MCF-7 (**C**) and MDA-MB-231 (**D**) cells transduced with lentiviral vectors carrying short hairpin RNA (shRNA) against PDIA1 or negative sequence after selection with puromycin in regarding to wild type cell line. Data represent the means ± SD with points for individual measurements, the representative photos are also presented. Statistical analysis was calculated using parametric one-way ANOVA followed by Dunnett’s multiple comparisons test (* *p* = 0.05, ** *p* = 0.01, *** *p* = 0.001, **** *p* = 0.0001).

MCF-7 and MDA-MB-231 cells pre-treated with bepristat 2a presented a significantly lowered ability to form colonies. This inhibitory activity of bepristat 2a was stronger against MDA-MB-231 than MCF-7 cells (Figure 1A,B). However, the silencing of PDIA1 in MCF-7 or MDA-MB-231 cells did not downregulate the clonogenicity of these cells. Furthermore, the transduction procedure itself diminished the clonogenicity of MCF-7/shN and MDA-MB-231/shN cells (Figure 1C,D).

### 2.6. Effects of PDIA1 Inhibition on Wound Healing and Migration of Breast Cancer Cells and Endothelial Cells

**Figure 2:**
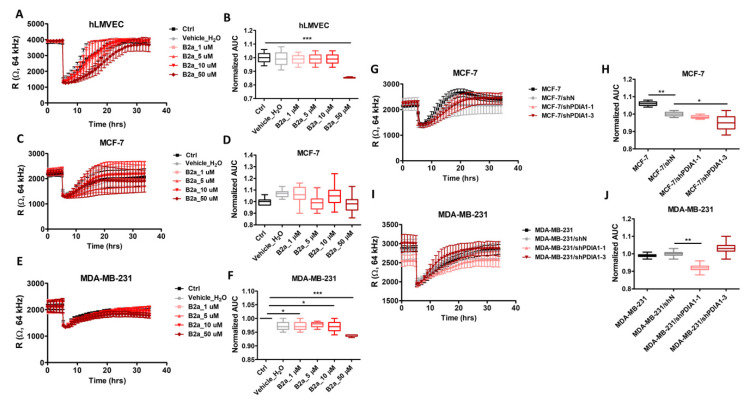
Effects of PDIA1 inhibition on wound-healing and migration of breast cancer cells and endothelial cells. ECIS Wound-Healing Assay of hLMVEC, MCF-7 and MDA-MB-231 cells and breast cancer sublines with silencing of PDIA1 (shN, shPDIA1-1 and shPDIA1-3). Real time tracings in hLMVECs (**A**), MCF-7 (**C**) and MDA-MB-231 (**E**) cell lines after addition of bepristat 2a at various concentrations (1, 10, 30 or 50 µM) and after PDIA1 silencing in MCF-7 (**G**) and MDA-MB-231 (**I**) cell lines. Area under the curve boxplots represent AUC quantitation of changes in migration rate of bepristat 2a-treated hLMVECs (**B**), MCF-7 (**D**) and MDA-MB-231 (**F**) cell lines versus non-treated controls as well as MCF-7 (**H**) or MDA-MB-231 (**J**) sublines transduced against PDIA1 (shPDIA1-1, shPDIA1-3) or wild type cells regarding to negative sequence (shN). The line graphs and AUC boxplots represent mean ± SD of three independent experiments. Statistical analysis was calculated using parametric one-way ANOVA followed by Dunnett’s multiple comparisons test (* *p* = 0.05, ** *p* = 0.01, *** *p* = 0.001).

In the Electric Cell-Substrate Impedance Sensing (ECIS)-based wounding assay for breast cancer cells and endothelial cells, the resistance of hLMVEC, MCF-7 and MDA-MB-231 cells untreated and after exposure to bepristat 2a (1–30 µM; Figure 2A,C,E) recovered rapidly to the state observed before electrical wounding. In hLMVECs, bepristat 2a at a 50 µM concentration induced a transient decrease in migration rate. After the incubation of MDA-MB-231 cancer cells with 50 µM of bepristat 2a, the recovery was less rapid than in the case of other concentrations, while MCF-7 cells migrated to the level observed before wounding similarly to control groups. Indeed, AUC for wound-healing response for hLMVEC and MDA-MB-231 but not for MCF-7 cells treated by bepristat 2a at 50 µM concentration was lower as compared to non-treated respective control cells (Figure 2B,D,F). Although the silencing of PDIA1 in MDA-MB-231 cells also induced changes in migration rate, this might be attributed to transduction effects. These results quantified by calculating the AUC wound-healing response for MCF-7 and MDA-MB-231 sublines (shPDIA1-1, shPDIA1-3) versus negative control (shN) are shown in Figure 2G–J.

### 2.7. Effect of PDIA1 Inhibition on Adhesion of Cancer Cells to the Endothelium

**Figure 3:**
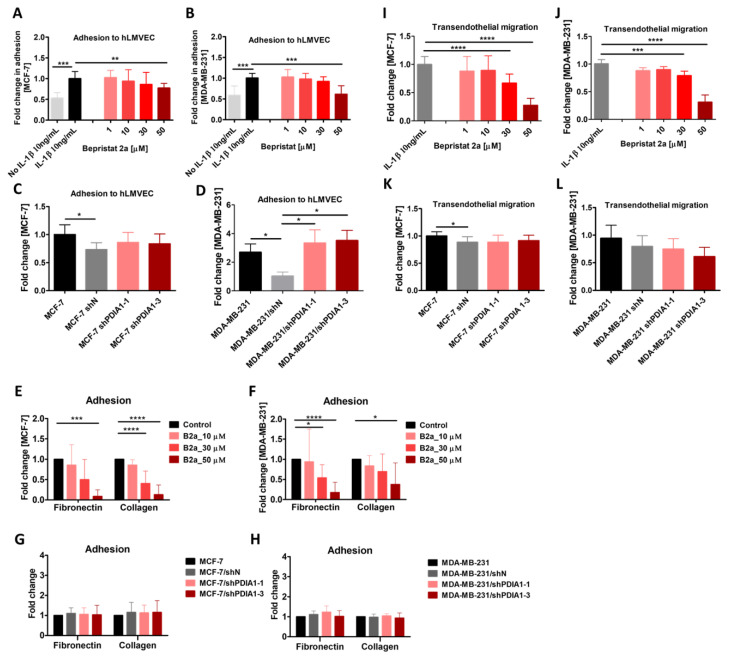
Effect of PDIA1 inhibition on adhesion of breast cancer cells to the endothelium, collagen type I and fibronectin and their transendothelial migration across hLMVEC monolayer. Effect of bepristat 2a on adhesion of MCF-7 (**A**,**E**) and MDA-MB-231 (**B**,**F**) cells to hLMVEC, collagen type I and fibronectin, respectively, and MCF-7 (**C**,**G**) and MDA-MB-231 (**D**,**H**) cells transduced with lentiviral vectors carrying short hairpin RNA (shRNA) against PDIA1 or negative sequence after selection with puromycin in regarding to wild type cell line. Transendothelial migration of MCF-7 (**I**) and MDA-MB-231 (**J**) cells treated with bepristat 2a and MCF-7 (**K**) and MDA-MB-231 (**L**) sublines with silenced PDIA1 expression across hLMVEC monolayers. Data represent mean ± SD of three independent experiments. Statistical analysis was performed using one-way ANOVA followed by Dunnett’s multiple comparisons test or nonparametric Kruskal–Wallis followed by Dunn’s multiple comparisons test (* *p* = 0.05, ** *p* = 0.01, *** *p* = 0.001, **** *p* = 0.0001).

As shown in Figure 3, the inhibition of PDIA1 by bepristat 2a resulted in decreased adhesion of both MCF-7 (Figure 3A) and MDA-MB-231 cells (Figure 3B) to endothelial monolayers in a concentration-dependent manner. Next, we examined the adhesion of MCF-7 and MDA-MB-231 cells after transduction with lentiviral vectors carrying shRNA against PDIA1. As shown in Figure 3, the downregulation of PDIA1 had no effect on MCF-7/shPDIA1-1 or MCF-7/shPDIA1-3 cell adhesion to hLMVECs compared to MCF-7/shN (Figure 3C). In the case of transduced MDA-MB-231 cells, MDA-MB-231/shN cells adhered much more weakly to the hLMVEC monolayer than did the MDA-MB-231 wild type, an effect interfering with the results of silencing in MDA-MB-231/shPDIA1-1 and MDA-MB-231/shPDIA1-3 (Figure 3D).

### 2.8. Effects of PDIA1 Inhibition on Adhesion of Cancer Cells to Fibronectin and Collagen Type I

The adhesion of MCF-7 and MDA-MD-231 cells to both ECM proteins was significantly inhibited by bepristat 2a used at a concentration of 50 µM, while 30 µM effectively inhibited the adhesion of MCF-7 cells to collagen and MDA-MB-231 cells to fibronectin (Figure 3E,F). In contrast to pharmacological blockade, the silencing of PDIA1 did not affect the adhesion of either cell line to either ECM protein (Figure 3G,H).

### 2.9. Effect of PDIA1 Inhibition on Transmigration of Cancer Cells across the Endothelium

As shown in Figure 3, bepristat 2a decreased the transendothelial migration of MCF-7 and MDA-MB-231 cancer cells in a concentration-dependent manner (Figure 3I,J). In the case of transduced MCF-7 cells, no inhibition was found in transmigration between shPDIA1-1 and shPDIA1-3 sublines and the control shN group (Figure 3K), but the negative control MCF-7/shN displayed an inhibitory effect. However, the MDA-MB-231/shPDIA1-3 subline showed non-statistically significant inhibition of transmigration compared to MDA-MB-231/shN (Figure 3L).

### 2.10. Effects of Exogenous PDIA1 and PDIA3 Proteins on Adhesion of Cancer Cells to Fibronectin and Collagen Type I and the Endothelium

**Figure 4:**
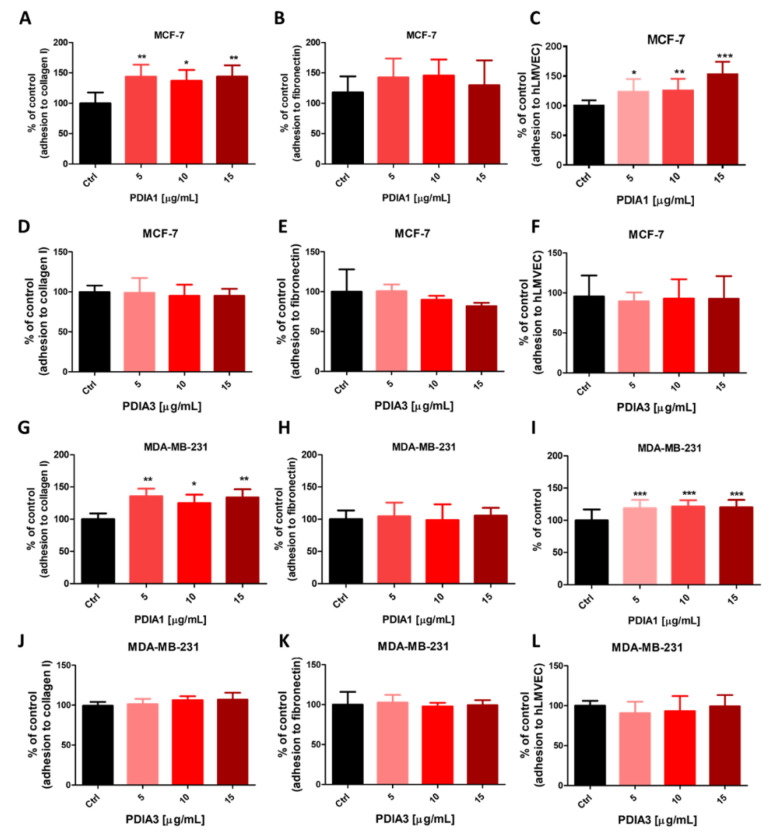
Effects of exogenous PDIA1 and PDIA3 on adhesive interaction between breast cancer cells and different substrates. Effect of exogenous proteins PDIA1 and PDIA3 on adhesion of MCF-7 (**A**–**F**) and MDA-MB-231 (**G**–**L**) cells to collagen type I, fibronectin and lung microvascular hLMVEC cells, respectively. Data represent mean ± SD of three independent experiments. Statistical analysis was performed using one-way ANOVA followed by Dunnett’s multiple comparisons test (* *p* = 0.05, ** *p* = 0.01, *** *p* = 0.001).

The exogenous PDIA1 protein increased the adhesion of MCF-7 and MDA-MB-231 cell lines to collagen and the hLMVEC monolayer (Figure 4A,C,G,I, respectively). However, the adhesion to fibronectin was not affected (Figure 4B,H). Moreover, MCF-7 and MDA-MB-231 cell adhesion to various substrates was not affected by exogenous PDIA3 (Figure 4D–F,J–L, respectively).

### 2.11. Effects of Thiol Blockers on Adhesion of Cancer Cells to Fibronectin and Collagen Type I

**Figure 5:**
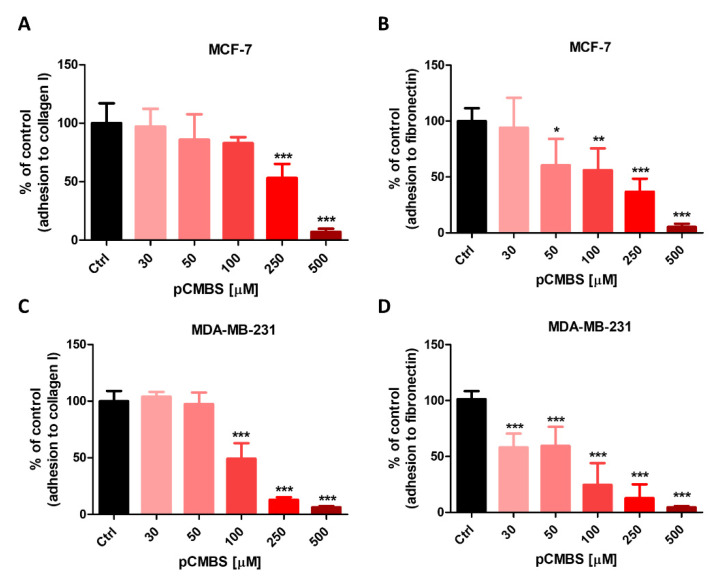
Effects of thiol blocker, pCMBS on adhesion of cancer cells to fibronectin and collagen type I. Effect of inhibiting ecto-sulfhydryls by p-chloromercuribenzene sulphonate (pCMBS) on adhesion of MCF-7 (**A**,**B**) and MDA-MB-231 (**C**,**D**) cells to collagen type I and fibronectin. Data represent mean ± SD of three independent experiments. Statistical analysis was performed using one-way ANOVA followed by Dunnett’s multiple comparisons test (* *p* = 0.05, ** *p* = 0.01, *** *p* = 0.001).

The cell-impermeable irreversible blocker of thiols and inhibitor of disulphide exchange agent pCMBS blocked both MCF-7 and MDA-MB-231 cell adhesion to collagen I and fibronectin in a concentration-dependent manner. Nearly complete inhibition of adhesion was observed at 500 µM pCMBS (Figure 5A–D).

### 2.12. Effect of PDIA1 Inhibition on ICAM-1 Expression

To verify whether bepristat 2a interfered with endothelial ICAM-1 expression, causing a decrease in cancer cell adhesion and transendothelial migration, hLMVECs were pre-treated with 10 ng/mL hIL-1β for 6 h, followed by treatment with bepristat 2a at various concentrations (1–50 µM) for 24 h. Bepristat 2a caused no changes in ICAM-1 expression (Supplementary Figure S6).

### 2.13. Effect of PDIA1 Inhibition on Mitochondrial Respiration

To exclude the observed effects of bepristat 2a on cancer cell adhesion and transendothelial migration being related to bioenergetic effects of this compound, mitochondrial respiration and glycolysis were analysed. In hLMVECs, bepristat 2a induced no changes in mitochondrial respiration (OCR) at concentrations of 1, 5, 10 or 30 µM, but a slight, nonsignificant inhibitory effect of bepristat 2a on mitochondrial respiration was visible at a concentration of 50 µM (Supplementary Figure S7). In MCF-7 and MDA-MB-231 cells, incubation with bepristat 2a for 24 h had no effect on mitochondrial respiration, and only a slight, nonsignificant decrease in OCR value was seen after 50 µM bepristat 2a.

### 2.14. Content of Integrins in MDA-MB-231, MCF-7 and hLMVEC Cell Lines

As shown in Table 4, β1 integrin was the most common and highly expressed integrin in all three cell types. In hLMVECs, as well as β1 integrin, β3, β4, β5, α2, α3, α5, α6, αV integrins were detected. Integrins α2, αV and α5 were also abundant but displayed lower expression levels compared with integrin β1. In MCF-7 cells, the integrin α2 level was similar to β1 integrin. In MDA-MB-231, integrins α2, α3, α6 and β4 were high, but their levels were lower than integrin β1.

## 3. Discussion

Adhesion of cancer cells to the endothelium and their subsequent transendothelial migration represent important steps in metastasis, and PDIA1 expression has been suggested to promote metastasis [10,31,32]. Here, we provide insight into the mechanisms involved and suggest that PDIA1 regulates the adhesion and transendothelial migration of breast cancer cells via disulphide re-arrangement of ecto-sulfhydryls, a mechanism previously described for integrin-mediated adhesion of leukocytes and platelets [7,26,27,28]. We confirmed the role of PDIA1 in regulating the cell cycle and apoptosis, which was translated into a noticeable anti-cancer effect in the colony formation assay but not a substantial anti-proliferation effect in the classical assays. Altogether, our results showed that, apart from the tumorigenic role of intracellular PDI in the ER [33], PDIA1 played a vital role in regulating the adhesion of cancer cells and their transendothelial migration. The latter effects could be attributed to the extracellular PDIA1 function of regulating integrin-mediated adhesion and subsequent transendothelial migration of breast cancer cells, but not to the PDIA1-dependent regulation of ICAM-1 expression or changes in cellular bioenergetics in breast cancer or endothelial cells.

In the present work, to assess the functional role of PDIA1, we used two approaches: selective inhibition of PDIA1 by bepristat 2a (IC_50_ 2.1 µM, Table 3) and silencing of PDIA1 in breast cancer cells by various silencing shPDIA1 hairpins. Although the major results were consistent in showing that the inhibition of PDIA1 disrupted the cell cycle and diminished the transendothelial migration of breast cancer cells, the effects of pharmacological inhibition by bepristat 2a were more substantial compared with the silencing of PDIA1 in cancer cells. These results indicate that the silencing of cancer cells may not necessarily provide a definite clarification as to the mechanisms involved. In fact, it might well be that silencing PDIA1, a key cellular enzyme, in rapidly proliferating cells could induce adaptive changes in the cancer cell proteome. That phenomenon is known in cancer cell biology; silencing approaches might trigger an unwanted resistance response for the cells to cope with the hostile perturbation [34,35]. Moreover transduction procedure did not result in a 100% downregulation of PDIA1 (Figure S1 and Table 2) so PDIA1 that stayed functional in cancer cells could be still partially active. 

As a pharmacological tool to inhibit PDIA1, we chose bepristat 2a because most of the other inhibitors of PDI identified in the last decade interact with the catalytic cysteines of PDI and are not selective towards PDI isoforms. However, bepristat 2a targets the substrate-binding domain of PDI, making it selective against PDIA1 and not against other thiol isomerases [36], as also confirmed here (Table 3). Previously, bepristats have been shown to impair platelet accumulation at sites of vascular injury in an in vivo model of thrombus formation [36], a response dependent on PDIA1, as confirmed in other studies [37]. Here, we have demonstrated for the first time, to our knowledge, that bepristat 2a prevented the adhesion of MCF-7 and MDA-MB-231 cells to collagen, fibronectin and endothelium, and impaired cancer cell transendothelial migration. A similar anti-adhesive effect was shown by pCMBS, a membrane-impermeant thiol blocker. Moreover, we showed that human protein PDIA1, but not PDIA3, increased cancer cell adhesion to the endothelial monolayer and collagen type I. Altogether, our results provide evidence that PDIA1 is important in regulating cancer cell adhesion to ECM proteins and endothelium by disulphide re-arrangement of ecto-sulfhydryls [26,27,28,37], in addition to its anti-cancer effects, attributed to cycle–cycle regulation by intracellular PDI.

Previous studies exploring the biological significance of PDIs have shown that several PDIs such as PDIA1, A3 and A6 play a significant role in cancer metastasis. Zhou et al. [25] reported that the expression of PDIA1 was significantly upregulated in colon cancer tissues compared with normal colon tissues and that its knockdown decreased cell proliferation and increased cell apoptosis in human cancer HT29 cells. Xia et al. [24] also found that PDIA1 promoted hepatocellular carcinoma cell growth, migration and invasion in vitro and tumour formation in vivo. Furthermore, the literature data indicate that high expression of PDIA1 plays an important role in diffuse glioma progression, correlated with high Ki-67 and more TP53 mutations [22]. Cell-surface PDIs have also been found to be associated with cancer invasion and metastasis [38]. Our results are compatible with previous work on the importance of intracellular PDIA1 in cancer progression. We have confirmed the involvement of PDIA1 in cycle–cycle regulation and apoptosis as suggested previously and extended previous results [10,22,24] by showing novel aspects of extracellular PDIA1-dependent regulation of tumourigenesis by affecting cancer cell interactions with endothelium.

To the best of our knowledge, for the first time using a proteomic approach, we characterised a full repertoire of PDIs in two human breast cancer cell lines: MCF-7 and MDA-MB-231. We demonstrated that PDIA1 was the most abundant isoform of PDIs in these cell lines, which was consistent with reports concerning the upregulation of PDIA1 in many cancer cell types [15]. Furthermore, PDIA1 was also detected in extracellular media taken from MCF-7 and MDA-MB-231 cancer cells. Interestingly, in primary lung microvascular endothelial cells, the level of PDIA1 was even higher than in breast cancer cells. Although we did not detect extracellular PDIA1 in media dedicated for hLMVECs in unstimulated cells, this cannot rule out PDIA1 being secreted by endothelial cells on injury or activation and localised to the surfaces of these cells, because extracellular PDIA1 has been reported to contribute to thrombus formation by catalysing the allosteric disulphide exchange in proteins such as tissue factor [39,40]. Thus, it is tempting to suggest that PDIA1, the major isoform of PDIs in these cells, released from cancer cells as well as hLMVECs, contributes to regulating cancer cell adhesion to endothelium by supporting integrin-mediated adhesion, in a manner similar to that seen in interactions between leukocytes [6,7] or platelets [41] and endothelium. Indeed, increasing evidence supports the notion that integrin activation depends on thiol/disulphide exchanges [26,42,43,44]. It remains to be established in further studies whether, apart from PDIA1, other PDIs including PDIA3, PDIA4 and PDIA6 displaying relative high expression in breast cancer cells and endothelium are also involved in regulating cancer cell interaction.

It has not been established here which type of integrin was involved in PDIA1-mediated cancer cell adhesion, because this topic requires additional mechanistical studies. However, we showed that in the presence of the membrane-impermeant thiol blocker pCMBS, cancer cell adhesion to collagen type I (a specific counter-ligand for integrin α2β1) and fibronectin (mediated for integrin α5β1) was effectively blocked. Another thiol blocker, DTNB, given at a non-toxic concentration (100 µM), also effectively inhibited MDA-MB-231 and MCF-7 cell adhesion to ECM proteins. These findings suggest that in PDIA1-mediated cancer cell adhesion, a major role can be ascribed to β1 integrin, which displayed the highest abundance of all integrins in breast cancer cells and pulmonary endothelial cells. A similar experimental approach using pCMBS and DTNB was adopted in studies of the role of thiols in platelet adhesion by Gofer-Dadosh [45] and Lahav et al. [46]. They showed that disulphide exchange is a necessary step in platelet adhesion to collagen, mediated by α2β1 integrin, indicating a specific involvement of surface PDIs in this process [27,28]. In our proteomic analysis, we observed relatively high levels of β1 integrin in endothelial lung microvascular cells and breast cancer cells. α2 was also highly expressed in breast cancer cell lines, while α5 was abundant in pulmonary endothelial cells. The role of β1 subunit–mediated adhesion seems most likely because in endothelial cells, β1 is linked with proper localisation of VE-cadherin and thus is crucial to cell–cell junction integrity [47]. On the other hand, high levels of β1 integrin in MDA-MB-231 cells correspond to their highly invasive phenotype and high efficiency of adhesion and migration [48], while MCF-7, as a non-invasive cell line, exhibited much lower levels of β1-subunit in our proteomic analysis. Integrin α2 forms heterodimers exclusively with β1 and has a widespread distribution in fibroblasts, endothelial cells, blood cells and epithelial cells [49,50]. Together with integrin α5β1, but not other members of the β1 subfamily, it plays a role in the maintenance of endothelial monolayer continuity in vitro [51]. In multiple types of cancer, integrin α2β1 promotes metastasis. In a mouse model of melanoma, integrin α2 inhibition resulted in diminished colon and breast cancer metastasis to the liver [52]. Monoclonal blocking antibodies for α2β1 integrin, but not αVβ3, inhibited adhesion to collagen type I and hLMVECs (unpublished data). Similar results were reported by Etoh et al. [53], who observed that blocking β1 and α2 integrins by monoclonal antibodies caused inhibition of the migration of melanoma cells. Haidari et al. [54] found that invasive MDA-MB-231 cells disrupted endothelial adherent junctions and promoted transendothelial migration, activating α2β1 heterodimer and mediating tyrosine phosphorylation of vascular endothelial cadherin. Accordingly, the role of β1 integrin in cancer cells (α2β1, α5β1) as well as in endothelial cells (α2β1, αVβ1) could represent a target for PDIA1 action, but the involvement of β3 integrin cannot be excluded [55,56]. Further studies are needed to set out which integrins are involved in PDI-mediated regulation of breast cancer cell adhesion and transmigration. During transendothelial migration, both tumour and endothelial cells undergo dynamic morphological and cytoskeletal changes, and integrins β1 and β3, present on cancer cells and the endothelium, associated with a variety of α chains, are active participants in the cell–cell interactions or cell-specific matrix protein (e.g., laminin)–cell interactions [57,58,59,60,61]. Thus, modulating the activation of various integrins on cancer and endothelial cells by a PDI-based approach seems to be more effective anti-metastatic strategy then the inhibition of a single integrin. Confirmation of this thesis in future studies will shed more light on that field.

Our previous results [29] clearly show that the suppression of cellular metabolism results in the inhibition of adhesion and transendothelial migration of cancer cells. In the current study, we found that bepristat 2a did not change mitochondrial respiration or glycolysis in cancer cells or endothelial hLMVECs. Among several known adhesion molecules, ICAM-1 is important in interactions between cancer and endothelial cells. We have previously established [29] that the adhesion of MDA-MB-231 cells to hLMVECs was significantly inhibited after blocking ICAM-1, but not VCAM-1 or E-selectin. However, in the present study, we found that inhibition of PDIA1 did not impact ICAM-1 expression. Altogether, these results excluded the existence of mechanisms of bepristat 2a activity comprising the effects on energy metabolism or ICAM-1. This, in turn, supports that adhesion and transendothelial migration of breast cancer cells is PDIA1 mediated. There are limitations of our study, including no clear-cut explanation of dissimilar effects of PDIA1 silencing and PDIA1 pharmacological inhibition, and lack of identification of a specific integrin involved. However, despite these shortcomings pharmacological evidence clearly demonstrates the regulatory role of PDIA1 and disulphide exchange in cancer cells adhesion and transendothelial migration.

## 4. Materials and Methods 

### 4.1. Cell Lines and Cell Culture

Human lung microvascular endothelial cell line (hLMVECs) was obtained from Cell Applications (San Diego, CA, USA). Human breast adenocarcinoma MCF-7 cell line was purchased from the European Collection of Authenticated Cell Cultures (ECACC, Salisbury, UK). MDA-MB-231 breast cancer cell line was commercially obtained from the American Type Culture Collection (ATCC, Rockville, Maryland, MD, USA). Lenti-X™ 293 T cell line (subclone of the transformed human embryonic kidney cell line; HEK 293) was purchased from Clontech (Clontech Laboratories, Inc., Takara Bio USA, Inc., Mountain View, CA, USA) and was used for lentiviral vector production. hLMVECs were maintained in microvascular endothelial cell growth medium (MECGM; Cell Applications, San Diego, CA, USA). MDA-MB-231 cells were cultured in RPMI 1640 (Hirszfeld Institute of Immunology and Experimental Therapy, Polish Academy of Sciences; HIIET PAS), supplemented with 2 mM L-glutamine and 10% (v/v) FBS (both from Sigma-Aldrich, Steinheim, Germany). MCF-7 cells were cultured in Eagle medium (HIIET PAS, Wroclaw, Poland), supplemented with 2 mM L-glutamine, 1% MEM non-essential amino acid solution, 0.8 mg/L of human insulin solution (all from Sigma-Aldrich, Steinheim, Germany), and 10% (v/v) FBS (Thermo Scientific, Waltham, MA, USA). Both culture media, RPMI 1640 and Eagle medium, contained antibiotics: 100 U/mL penicillin (Sigma-Aldrich, Steinheim, Germany), 100 μg/mL streptomycin (Polfa Tarchomin, Warsaw, Poland) and 0.25 μg/mL amphotericin B (Sigma-Aldrich, Steinheim, Germany). The Lenti-X™ 293 T cells were cultured in high-glucose Dulbecco’s Modified Eagle Medium (Gibco, Scotland, UK), supplemented with 10% (v/v) FBS (HyClone, GE Healthcare, Little Chalfont, UK) and 1 mM sodium pyruvate (Sigma-Aldrich, Steinheim, Germany). Cells were maintained in an incubator at 5% CO_2_ in air and 37 °C. Regularly, cells were used for two to nine passages after thawing. All cell lines were regularly tested for Mycoplasma (Lonza, Basel, Switzerland) contamination before cryopreservation and every 3 months.

### 4.2. Proteomic Studies

hLMVECs, MDA-MB-231 and MCF-7 wild type breast cancer cell lines, and their sublines (shN, shPDIA1-1 and shPDIA1-3) in three to five consecutive passages were seeded in equal amounts, grown until 90–95% confluent, detached using Accutase solution (Sigma-Aldrich, Steinheim, Germany) and washed twice with Dulbecco’s Phosphate-Buffered Saline (DPBS; Gibco, UK; 200 g, 5 min, room temperature [RT]). The material was frozen at −80 °C until analysis. The cell lysate was prepared for proteomic analysis according to Sitek et al. [62] with slight modifications. Briefly, 10 µg of proteins were reduced, alkylated and digested with trypsin. The resulting peptides were subjected to LC-MS/MS analysis (Mass Spectrometry Laboratory at the Institute of Biochemistry and Biophysics, Polish Academy of Sciences, Warsaw, Poland) [63]. The acquired MS/MS data were pre-processed with Mascot Distiller software (Matrix Science, London, UK), and a search was performed with the Mascot search engine against the human proteins. For the detection and semi quantitation of PDI isoforms and integrin isoforms, the exponentially modified protein abundance index (emPAI) calculation was performed [64,65]. The detailed procedures for proteomic studies are described in the Supplementary Materials and Methods.

### 4.3. Knockdown of PDIA1 in Human Breast Adenocarcinoma MDA-MB-231 and MCF-7

**Figure 6:**
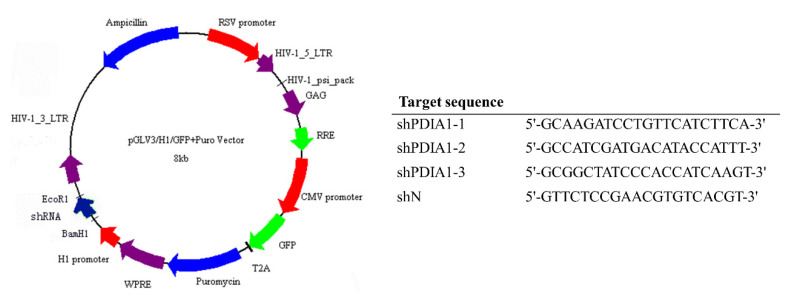
The scheme of lentiviral vector used for silencing of PDIA1 expression and sequences of short hairpin RNAs (shRNAs) cloned to the plasmid.

The third-generation lentiviral system consisting of pMDLg/pRRE, pRSV-Rev, pMD2.G (the plasmids were a gift from Didier Trono; Addgene plasmid #12251, 12253, 12259) and expression plasmids pGLV-H1-GFP-Puro (EZBiolab Inc., Carmel, IN, USA) were used to establish new MDA-MB-231 and MCF-7 cell lines with silenced expression of PDIA1. The expression plasmids encoded three different short hairpin RNA (shRNA) sequences designed for PDIA1 (shPDIA1 1–3). The control vector (used as a negative control) encoded a negative shRNA sequence (shN). The map of the expression plasmid and sequences of shRNA cloned to the plasmid is presented in Figure 6. Lentiviral vectors were produced using the Lenti-X™ 293 T cell line according to the protocol described by Rossowska et al. [66] and applied to human MDA-MB-231 and MCF-7 cell line transduction.

After transduction, the cell sublines MDA-MB-231/shPDIA1-1, MDA-MB-231/shPDIA1-2, MDA-MB-231/shPDIA1-3, MDA-MB-231/shN, MCF-7/shPDIA1-1, MCF-7/shPDIA1-2, MCF-7/shPDIA1-3 and MCF-7/shN were selected by culturing them in the growth medium for MDA-MB-231 or MCF-7 cell line supplemented with 10 μg/mL puromycin (Gibco, Scotland, UK) to obtain stable cell lines. The expression of PDIA1 in genetically modified cells was evaluated relative to wild type MDA-MB-231 and MCF-7 cell lines.

### 4.4. Real-Time PCR and Western Blot

PDIA1 expression in the obtained cell sublines was analysed using real-time PCR and Western blot assays. The detailed procedures for these assays are described in the Supplementary Materials and Methods.

### 4.5. Measurements of Bepristat 2a Effect on Insulin Reduction Catalyzed by PDIA1 and Other PDIs 

The protein disulfide isomerase activity was measured as an increase in disulfide bonds reduction in human insulin in the presence of DTT causing aggregation of its β-chain, a process that can be followed by turbidimetry. Briefly, the assay mixture for 96-well plates was prepared by dissolving in 0.1 mM phosphate buffer (pH 7.6 for activity testing of PDIA1, PDIA3, PDIA4 or PDIA6 and pH 8.0 for PDIA17), 6 µg/mL (96 nM) of PDIA1 (recombinant protein MBS9422429, human, full length, 18-508aa His-tag, E.coli; (MyBioSource, San Diego, CA, USA), PDIA3 (recombinant protein MBS203583, 25-505aa, human, His-tag, E.coli, MyBioSource), PDIA4, (recombinant protein MBS2902080, 21-645aa, human, His-tag, E.coli, MyBioSource), PDIA6 (recombinant protein MBS144267, 20-440aa, human, His tag, E.coli, MyBioSource) or PDIA17 (recombinant protein ab64013, human, full length, *E. coli*, (Abcam, Cambridge, UK), 2 mM EDTA and 0.08 mM DTT (D9779 Sigma-Aldrich, Steinheim, Germany). Stock solutions of bepristat 2a were freshly prepared in DMSO and subsequently diluted to keep the final DMSO concentration in assay mixture below 1%. Calculated amounts of compounds solutions were added into test wells and the reaction was started by addition of insulin (insulin 91077C, human recombinant; Sigma-Aldrich, Steinheim, Germany) and 0.08 mM of DTT. The final concentration of insulin and DTT in assay mixture was 0.15 and 0.16 mM, respectively. The reaction rate was monitored at 650 nm on a Microplate Reader Infinite M1000 PRO for 60 min at 37 °C. Turbidity values for the wells containing only the PDI isoform tested (background values) was subtracted from the turbidity values of the wells containing PDI isoform tested + bepristat 2a. The inhibition of PDI isoform’s catalytic activity in the presence of bepristat 2a was calculated by the following formula: enzyme inhibition (%) = [1 − (OD[bepristat + PDI + DTT] − OD[DTT])/(OD[PDI + DTT] − OD[DTT])] × 100%, where OD means optical density.

### 4.6. SRB Assay

The proliferation rate of the cells with silenced expression of PDIA1 was measured using SRB assay (see Supplementary Materials and Methods for more details).

### 4.7. MTS Assay

The effect of bepristat 2a on endothelial and breast cancer cell viability was determined by MTS assay (see Supplementary Materials and Methods for more details).

### 4.8. Caspase 3/7 Activity

Measurements of the caspase 3/7 activity were performed with fluorogenic assay as described in the Supplementary Materials and Methods.

### 4.9. Cell Cycle Analysis

Flow cytometry was used to assess the effect of PDIA1 inhibition on the cell cycle progression of MDA-MB-231 and MCF-7 cells. This procedure is described in the Supplementary Materials and Methods.

### 4.10. Long-Term Colony Formation Assay

The long-term colony formation assay was performed as previously described by Pawlak [67]. MDA-MB-231 and MCF-7 cell lines and their sublines with PDIA1 silenced (shN, shPDIA1-1 and shPDIA1-3) were seeded on 6-well plates in triplicate (Corning, Kennebunk, ME, USA) for 9-day colony formation assay in the appropriate culture medium, at a density of 1 × 10^3^ cells/well and 5 × 10^2^ cells/well for MDA-MB-231 and MCF-7, respectively, on the day of subculturing. To confirm a long-lasting reduction in clonogenic potential by bepristat 2a hydrochloride (Sigma-Aldrich, Steinheim, Germany), 24 h before adding the tested compound, MDA-MB-231 and MCF-7 cells were seeded on six-well plates (Corning, Kennebunk, ME, USA) at a density of 0.5 × 10^6^ cells/well. The cells were treated with bepristat 2a at concentrations of 10, 30 and 50 µM and collected after 24 h of incubation. The viable cells were counted and seeded in triplicate at the densities described on six-well plates. After 9 days, the colonies were fixed and stained with 1% crystal violet/methanol (Sigma-Aldrich), documented with a Sony Alpha 300 camera (Sony, Tokyo, Japan) and counted manually using ImageJ 1.47 software (National Institutes of Health, Bethesda, MD, USA). The colony number was calculated.

### 4.11. Electric Cell-Substrate Impedance-Sensing Assays

To measure the migratory potential of cancer cells with silenced PDIA1 or the impact of bepristat 2a on the migration rate of hLMVEC, MDA-MB-231 and MCF-7 cells, real-time quantitative wound-healing assays were performed and the 96W1E+ ECIS arrays (Applied BioPhysics, Troy, NY, USA) were used. Before the cell seeding procedure, the 96W1E+ plate was pre-treated for 10 min with 10 mM L-cysteine (Sigma-Aldrich, Steinheim, Germany) at RT and washed twice by ultrapure water. After washing, 200 μL of MECGM (for hLMVECs), RPMI1640 (for MDA-MB-231) or Eagle medium (for MCF-7) were added to each well to check resistance (Ω), capacitance (μF) and impedance (Ω) basal values. Then, the cells were added (hLMVEC and MDA-MB-231 cell lines were seeded at a density of 3 × 10^4^ per well, MCF-7 cell lines at a density of 6 × 10^4^ cells per well) with MECGM, RPMI1640 or Eagle medium, respectively, in a final volume of 300 μL. Resistance, capacitance and impedance values were recorded at frequencies from 250 Hz to 64 kHz (250, 500, 1000, 2000, 4000, 8000, 16,000, 32,000 and 64,000 Hz) using the multiple frequency time mode. The cell-free wells served as a negative control to provide the baseline changes in impedance for all experiments. Wounding was performed by applying an alternating current of 1600 µA, 64 kHz for 30 s, killing the cells on the surface of the electrode, which resulted in an abrupt drop in impedance to values like those of a cell-free electrode. The dead cells were washed away and fresh medium was added (for sublines with PDIA1 silenced) or medium containing bepristat 2a at various concentrations (1, 10, 30 or 50 µM). The healing process was measured continuously as cells migrated onto the electrode. The area under the curve (AUC) was quantified and normalized to untreated control or negative control (shN). The experiment was performed in a humidified 5% CO_2_ incubator at 37 °C and was repeated three times in seven technical replicates.

### 4.12. Adhesion Assay

#### 4.12.1. Cancer Cell Adhesion to Lung Microvascular Endothelial Cells

The hLMVECs were transferred into 96-well plates (seeding density 2.5 × 10^4^ cells/well) and left to grow to confluence for 48–72 h. Endothelial cells were stimulated with 10 ng/mL hIL-1β (Cell Signalling Technology, Leiden, The Netherlands) for 6 h. To determine the contribution of PDIA1 in cancer cell adhesion to the endothelial monolayer, pharmacological inhibitor, bepristat 2a or human PDIA1 protein and MDA-MB-231 and MCF-7 cell lines with silencing of PDIA1 (shN, shPDIA1-1 and shPDIA1-3) were used. Before use for the cell adhesion assay, MDA-MB-231 or MCF-7 cells were stained with Calcein-AM (BD Pharmingen, San Jose, CA, USA) according to the manufacturer’s instruction. Then, cancer cells were left to adhere to the endothelial layer with or without bepristat 2a at various concentrations (1, 10, 30 or 50 µM) for 30 min at 37 °C. Non-adherent cells were gently washed twice with DPBS.

In all assays, the attached cells were counted in six to nine randomly selected visual fields for each well. Pictures were taken using the CQ1 image cytometer (Yokogawa, Tokyo, Japan). Experiments were conducted three times in five to six technical replicates. The images were analysed using Columbus v. 2.4.2 software (Perkin Elmer, Waltham, MA, USA). The mean inhibition of adhesion for visual fields was calculated by using the equation: % of control = [number of adhered cells in treated samples/number of adhered cells in control group] × 100%.

#### 4.12.2. Cancer Cell Adhesion to Fibronectin or Collagen Type I

MDA-MB-231 and MCF-7 cell lines and their sublines (shN, shPDIA1-1 and shPDIA1-3) were seeded at a density of 5 × 10^5^ cells/well in the appropriate culture medium with 10% (v/v) FBS in 96-well plates (Corning, Kennebunk, ME, USA). The next day, the culture medium was changed to medium with 5% (v/v) FBS alone (all cell lines) or along with 10, 30 and 50 µM bepristat 2a hydrochloride (only wild type MDA-MB-231 and MCF-7 cell lines). On the same day, 96-well plates (Thermo Fisher Scientific, Waltham, MA, USA) were coated with collagen type I or fibronectin, both at a concentration of 10 μg/mL (both from Sigma-Aldrich, Steinheim, Germany), and incubated at 4 °C overnight. After 24 h, the plates were blocked with 1% BSA (Sigma-Aldrich, Steinheim, Germany) in TSM buffer (20 mM Tris-HCl pH 8.0, 150 nM NaCl, 1 mM CaCl2, 2 mM MgCl_2_; HIIET) for 30 min at 37 °C. On the same day, cells were nonenzymatically detached, centrifuged, counted and suspended in TSM buffer. Next, the 5 × 10^4^ cells/well were seeded in the coated plates and incubated at 37 °C for 1 h. After incubation, the cells were stained with 0.2% (w/v) crystal violet dissolved in methanol. The plate was incubated at 4 °C for 30 min. The absorbance was measured at 570 nm using a Synergy H4 reader and GEN5 software. The experiment was repeated at least three times.

The effect of the sulfhydryl blocker on cancer cell adhesion to fibronectin and collagen type I was measured using a membrane non-penetrating reagent, p-chloromercuribenzene sulphonate (pCMBS; Toronto Research Chemicals, Toronto, Ontario, Canada). Human PDIA1 protein (Pure Biologics, Wroclaw, Poland) was used to examine the effect of exogenous PDIA1 on both cancer cells’ adhesion to extracellular matrix (ECM) proteins. Briefly, after overnight coating with collagen type I or fibronectin, the 96-well plates were blocked with 1% BSA in PBS for 30 min at 37 °C. Before adding MCF-7 and MDA-MB-231 cells into the cell adhesion assay, cancer cells were stained with Calcein-AM (BD Pharmingen, San Jose, CA, USA), and pCMBS was added 15 min before introduction of MCF-7 and MDA-MB-231 to the adhesive surfaces. Then, cancer cells were left to adhere to collagen I and fibronectin for 30 min at 37 °C. Non-adherent cells were gently washed twice with DPBS, and attached cells were counted in six randomly selected visual fields for each well using the CQ1 image cytometer. The experiment was conducted three times in six technical replicates.

### 4.13. Transendothelial Migration Assay

Cell migration was assayed in 24-well, 6.5-mm internal diameter Transwell plates (8.0-μm pore size; BD Pharmingen, San Jose, CA, USA). hLMVECs (seeding density 5 × 10^4^ cells/insert) were first cultured for 72 h on the upper side of the filter with complete medium. After confluent monolayer formation, the hLMVECs were pre-treated with 10 ng/mL hIL-1β for 6 h. Then, MDA-MB-231 or MCF-7 cells (wild type or their sublines with silenced PDIA1, each 5 × 10^4^ per well) were placed into upper chambers in the presence or absence of bepristat 2a at various concentrations (1, 10, 30 or 50 µM). Lower chambers were filled with EBM-2 serum-free medium (Lonza, Basel, Switzerland) containing stromal cell-derived factor 1α (SDF-1, 100 ng/mL; Sigma-Aldrich, Steinheim, Germany). After 24 h of co-culture, hLMVEC monolayers and non-migrating cancer cells on the upper surface of the membrane were removed, and migrated cancer cells on the undersides of the Transwell membranes were detached and stained by Calcein-AM−Accutase solution for 60 min. The cell number was determined by measuring the fluorescence using a plate reader. The experiments were performed three times with three technical replicates.

### 4.14. Expression of ICAM-1

Immunostaining was performed to verify whether bepristat 2a interferes with surface ICAM-1 expression in hLMVECs (see Supplementary Materials and Methods for detailed method description).

### 4.15. Analysis of Cellular Bioenergetics by Extracellular Flux Technology

To determine whether the effects of bepristat 2a on the interactions between MCF-7 and MDA-MB-231 and hLMVECs were correlated with their effects on the oxygen consumption rate (OCR) and extracellular acidification rate (ECAR), a Seahorse XF technique was used (a detailed procedure for this method is provided in the Supplementary Materials and Methods).

### 4.16. Statistical Analysis

All results are presented as the mean value ± standard deviation (SD). Statistical analysis was performed using GrapPad Prism 7 (San Diego, CA, USA). The assumptions of analysis of variance (ANOVA) were checked using the Shapiro–Wilk normality test and the Brown–Forsythe test. If the assumptions of the parametric test were fulfilled, a one-way ANOVA test followed by Dunnett’s multiple comparisons test was run. If they were not met, the nonparametric Kruskal–Wallis test was performed, followed by Dunn’s multiple comparisons test. Specific tests used for data analysis are listed in the figure legends. Differences with *p*-values lower than 0.05 were considered statistically significant.

## 5. Conclusions

The current study is the first to demonstrate that extracellular PDIA1 regulates breast cancer interactions with lung microvascular endothelial cells during metastasis. Described mechanism involves disulphide exchange and most likely activation of integrin-mediated cancer cell adhesion and transmigration. Whether other isoforms of PDIs also regulate this process similarly to multiple PDIs involved in activating platelet integrins [40,46,68,69] remains to be established. Finally, our results suggest that the inhibition of extracellular PDIA1 or other PDIs represents an interesting target for anti-metastatic treatment that warrants further exploration. Changes in endothelial function have an impact on the development and progression of almost all diseases. Thus, expanding the knowledge of endothelial repertoire of PDIs and understanding specific molecular mechanisms used by cancer cells in dynamic interactions with endothelial cells will help to identify promising molecular targets for anticancer therapy.

## Figures and Tables

**Table 1:** Relative content of protein disulphide isomerases (PDIs) in hLMVEC, MCF-7 and MDA-MB-231 cell lines.

PDI Isoform	hLMVEC [Mean ± SD]	MCF-7 [Mean ± SD]	MDA-MB-231 [Mean ± SD]
**PDIA1**	10.297 ± 1.374	8.987 ± 2.895	7.220 ± 0.907
**PDIA3**	7.313 ± 0.918	7.920 ± 4.092	5.063 ± 0.903
PDIA4	4.020 ± 0.000	4.387 ± 2.620	2. 943 ± 1.101
**PDIA5**	0.233 ± 0.085	0.235 ± 0.120	not detected
**PDIA6**	3.503 ± 0.231	2.807 ± 0.512	1.833 ± 0.006
**PDIA9**	2.160 ± 0.000	2.630 ± 1.250	2.190 ± 0.460
**PDIA10**	0.660 ± 0.087	1.380 ± 0.314	0.663 ± 0.179
**PDIA11**	0.420 ± 0.104	0.850 ± 0.678	0.567 ± 0.319
**PDIA12**	0.130 ± 0.104	0.130 ± 0.000	0.130 ± 0.000
**PDIA13**	0.177 ± 0.095	not detected	0.130 ± 0.071
**PDIA14**	0.110 ± 0.000	0.240 ± 0.000	not detected
**PDIA15**	3.443 ± 1.564	1.670 ± 0.962	1.783 ± 0.365
**PDIA16**	1.217 ± 0.266	0.440 ± 0.173	0.540 ± 0.000
**PDIA17**	not detected	6.117 ± 1.58	0.230 ± 0.000
**PDIA18**	not detected	1.640 ± 0.896	not detected
**PDIA19**	0.380 ± 0.104	0.260 ± 0.000	0.100 ± 0.000

Semi-quantitative data (emPAI) for each detected PDI isoform in lysates from human lung microvascular endothelial cells (hLMVEC) and breast cancer MCF-7 and MDA-MB-231 cell lines. Data represent the means ± SD from three consecutive passages. emPAI was calculated based on the number of observed peptides per proteins normalized by the theoretical number of peptides subtracted from LC-MS/MS data using MascotTM (Matrix Sciences, London, UK).

**Table 2:** Relative content of PDIs (emPAI) in MCF-7 and MDA-MB-231 cell lines after PDIA1 silencing.

PDI Isoform	MCF-7 Cells				MDA-MB-231 Cells			
	Wild Type	shN	shPDIA1-1	shPDIA1-3	Wild Type	shN	shPDIA1-1	shPDIA1-3
**PDIA1**	9.032 ± 0.846	8.845 ± 1.853	0.163 ± 0.085	2.043 ± 0.611	5.002 ± 0.508	2.000 ± 1.185	4.348 ± 2.359	1.145 ± 0.382
**PDIA3**	7.454 ± 1.309	7.700 ± 2.079	7.388 ± 1.078	7.597 ± 1.594	5.366 ± 1.523	6.653 ± 1.631	5.418 ± 0.979	7.565 ± 1.292
**PDIA4**	5.448 ± 0.879	5.190 ± 0.846	5.630 ± 0.664	2.707 ± 0.320	3.698 ± 0.947	4.035 ± 0.424	4.270 ± 0.757	3.508 ± 0.622
**PDIA5**	0.070 ± 0.000	0.150 ± 0.080	0.150 ± 0.080	0.070 ± 0.000	not detected	0.070 ± 0.000	0.070 ± 0.000	0.070 ± 0.000
**PDIA6**	2.986 ± 0.646	3.110 ±00.334	3.030 ± 0.404	2.790 ± 0.191	2.576 ± 0.490	3.048 ± 0.638	2.966 ± 0.538	3.200 ± 0.347
**PDIA9**	3.654 ± 0.747	3.410 ± 0.589	2.940 ± 0.323	2.80 ± 0.606	2.018 ± 0.487	2.088 ± 0.756	2.118 ± 0.566	2.350 ± 0.784
**PDIA10**	1.850 ± 0.565	1.950 ± 0.533	1.848 ± 0.125	1.173 ± 0.231	0.604 ± 0.252	0.878 ± 0.229	0.776 ± 0.287	0.605 ± 0.187
**PDIA11**	0.600 ± 0.110	0.868 ± 0.23	0.868 ± 0.243	0.420 ± 0.104	0.580 ± 0.291	0.435 ± 0.090	0.672 ± 0.254	0.535 ± 0.183
**PDIA12**	0.130 ± 0.000	0.130 ± 0.000	0.130 ± 0.000	0.130 ± 0.000	0.130 ± 0.000	0.130 ± 0.000	0.137 ± 0.012	0.130 ± 0.000
**PDIA13**	not detected	not detected	0.080 ± 0.000	not detected	not detected	not detected	0.080 ± 0.000	0.080 ± 0.000
**PDIA14**	0.143 ± 0.065	0.175 ± 0.092	0.173 ± 0.072	not detected	0.110 ± 0.000	0.110 ± 0.000	0.130 ± 0.000	0.110 ± 0.000
**PDIA15**	2.928 ± 0.544	2.793 ± 0.313	2.738 ± 0.594	2.220 ± 0.43	2.128 ± 0.396	2.330 ± 0.850	2.060 ± 0.477	2.185 ± 0.259
**PDIA16**	1.094 ± 0.252	0.933 ± 0.340	1.255 ± 0.230	0.440 ± 0.173	0.765 ± 0.488	0.558 ± 0.274	1.250 ± 0.430	0.598 ± 0.534
**PDIA17**	10.732 ± 1.024	9.200 ± 2.680	11.320 ± 2.096	8.420 ± 2.399	0.423 ± 0.167	1.130 ± 0.565	0.697 ± 0.537	0.375 ± 0.205
**PDIA18**	1.554 ± 0.740	1.305 ± 0.576	1.305 ± 0.576	1.253 ± 0.595	not detected	not detected	0.240 ± 0.000	0.240 ± 0.000
**PDIA19**	0.130 ± 0.057	0.193 ± 0.081	0.305 ± 0.900	0.133 ± 0.029	0.050 ± 0.000	0.083 ± 0.029	0.078 ± 0.049	0.063 ± 0.025

Semi-quantitative data for each detected PDI isoform (emPAI) in lysates from breast cancer MCF-7 and MDA-MB-231 cell lines after PDIA1 silencing. Data represent the means ± SD from minimum of 3 consecutive passages (MCF-7, WT—*n* = 5, shN—*n* = 4, shPDIA1-1—*n* = 4, shPDIA1-3—*n* = 3; MDA-MB-231, WT—*n* = 5, shN—*n* = 4, shPDIA1-1—*n* = 5, shPDIA1-3—*n* = 4). emPAI was calculated based on the number of observed peptides per proteins normalized by the theoretical number of peptides subtracted from LC-MS/MS data using MascotTM (Matrix Sciences, London, UK).

**Table 3:** Insulin reduction by PDIA1, PDIA3, PDIA4, PDIA6 and PDIA17 in the presence of bepristat 2a.

Bepristat 2a [µM]	*Enzymatic Activity, % of Initial*				
	PDIA1	PDIA3	PDIA4	PDIA6	PDIA17
200	*20 ± 6*	*36 ± 5*	*80 ± 2*	*69 ± 4*	*46 ± 8*
20	*42 ± 2*	*86 ± 4*	*89 ± 3*	*99 ± 2*	*95 ± 3*
2	*63 ± 2*	*103 ± 4*	*102 ± 2*	*95 ± 3*	*98 ± 3*
0.2	*72 ± 3*	*105 ± 2*	*100 ± 2*	*103 ± 2*	*100 ± 2*
0.02	*90 ± 3*	*not detected*	*not detected*	*not detected*	*not detected*
IC_50_	2.1	127	>200	>200	≥200

Recombinant PDIA1, PDIA3, PDIA4, PDIA6 and PDIA17 enzymes were incubated with bepristat 2a at various concentrations (0.02–200 µM) for 60 min at 37 °C, and then the activity of enzymes was monitored in insulin turbidimetric assay. Data represent the means ± SD obtained from 3 independent experiments. Effects of bepristat 2a inhibition on PDI isoforms are expressed as IC_50_ values.

**Table 4:** Content of integrins in hLMVEC, MCF-7 and MDA-MB-231 cell lines.

Integrin	hLMVEC [Mean ± SD]	MCF-7 [Mean ± SD]	MDA-MB-231 [Mean ± SD]
**β1**	0.853 ± 0.133	0.250 ± 0.180	0.950 ± 0.499
**β3**	0.100 ± 0.000	not detected	not detected
**β4**	0.050 ± 0.036	0.110 ± 0.000	0.347 ± 0.119
**β5**	0.050 ± 0.000	0.050 ± 0.000	0.050 ± 0.000
**α2**	0.430 ± 0.000	0.180 ± 0.000	0.407 ± 0.151
**α3**	0.157 ± 0.075	not detected	0.520 ± 0.115
**α5**	0.477 ± 0.064	not detected	0.080 ± 0.000
**α6**	0.183 ± 0.100	not detected	0.270 ± 0.200
**αV**	0.387 ± 0.050	0.040 ± 0.000	0.103 ± 0.060

Semi-quantitative data (emPAI) for integrins detected in lysates from human lung microvascular endothelial cells (hLMVEC) and breast cancer MCF-7 and MDA-MB-231 cell lines. Data represent the means ± SD from three consecutive passages. emPAI was calculated based on the number of observed peptides per proteins normalized by the theoretical number of peptides subtracted from LC-MS/MS data using MascotTM (Matrix Sciences, London, UK).

## Supplementary Materials

The following are available online at https://www.mdpi.com/2072-6694/12/10/2850/s1, **Figure S1**: The activity of the shPDIA1 sequence in a stable transduced breast cancer cell lines, **Figure S2**: The effect of PDIA1 inhibition by bepristat 2a on hLMVEC, MCF-7 and MDA-MB-231 cells viability assessed after 24 and 48 h, **Figure S3**: The effect of PDIA1 inhibition on caspase 3/7 activity in breast cancer cells, **Figure S4**: The effect of PDIA1 inhibition on cell cycle distribution of breast cancer cells, **Figure S5**: The effect of PDIA1 inhibition on cell cycle distribution of breast cancer cells, **Figure S6**: The effect of PDIA1 inhibition on ICAM-1 expression. **Figure S7**: The effect of PDIA1 inhibition on mitochondrial respiration. **Figure S8**: The whole western blot figures.
