# Supplementary material for: Protein Disulphide Isomerase A1 Is Involved in the Regulation of Breast Cancer Cell Adhesion and Transmigration via Lung Microvascular Endothelial Cells
Source: Cancers (Basel). 2020 Oct 2;12(10):2850. doi: 10.3390/cancers12102850 (PMC7601413; doi:10.3390/cancers12102850)
Supplement: Supplementary file 1 [file cancers-12-02850-s001.zip › Supplementary_proof/cancers-931897-Supplementay figureS8-WB-image.pdf]

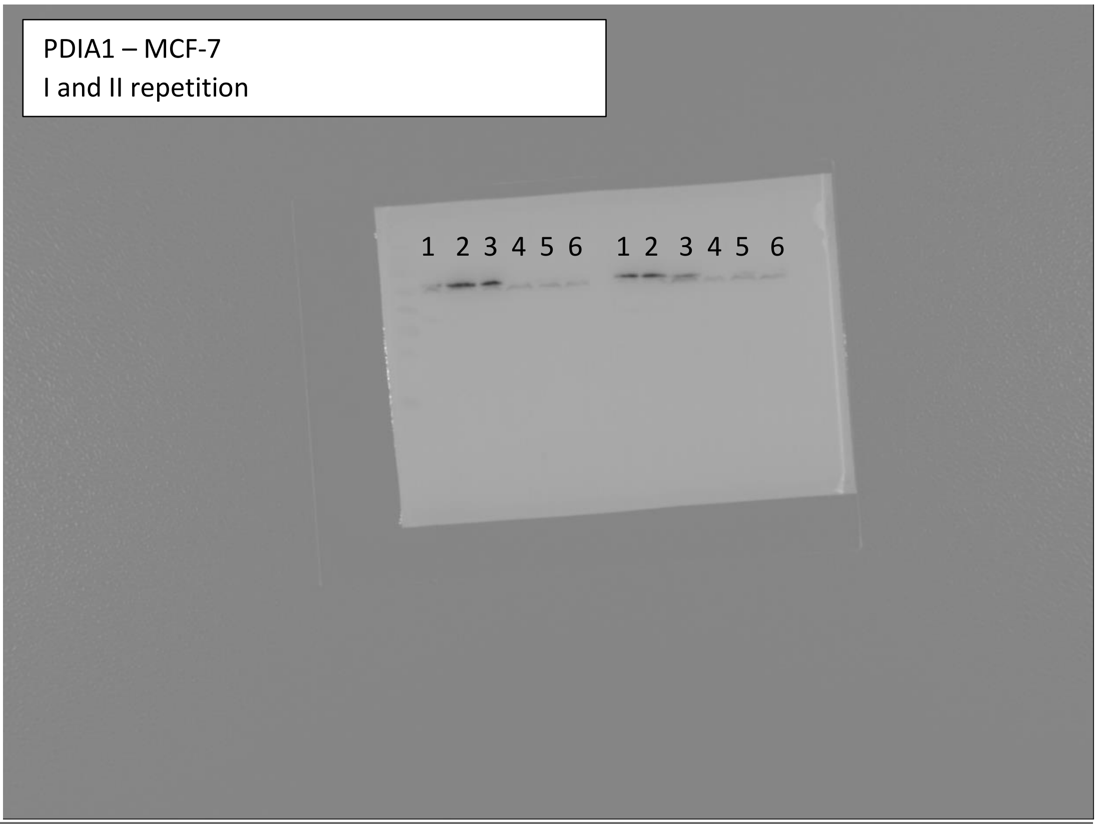

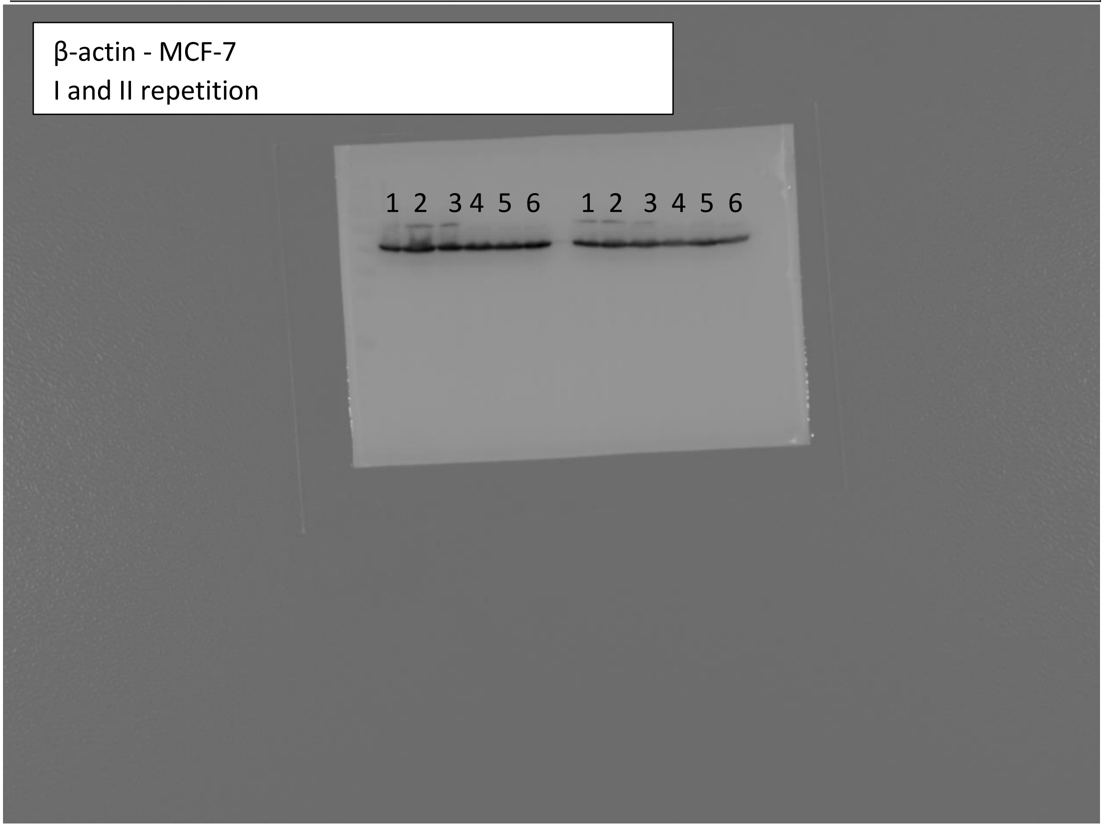

Fig. 1. PDIA1 and β-actin level in MCF-7 cells – first and second independent experiment.
1 – MCF-7 wt; 2 - MCF-7 shN; 3 - MCF-7 shGAPDH; 4 – MCF-7 shPDIA1-1; 5 - MCF-7 shPDIA1-2; 6 - MCF-7 shPDIA1-3.

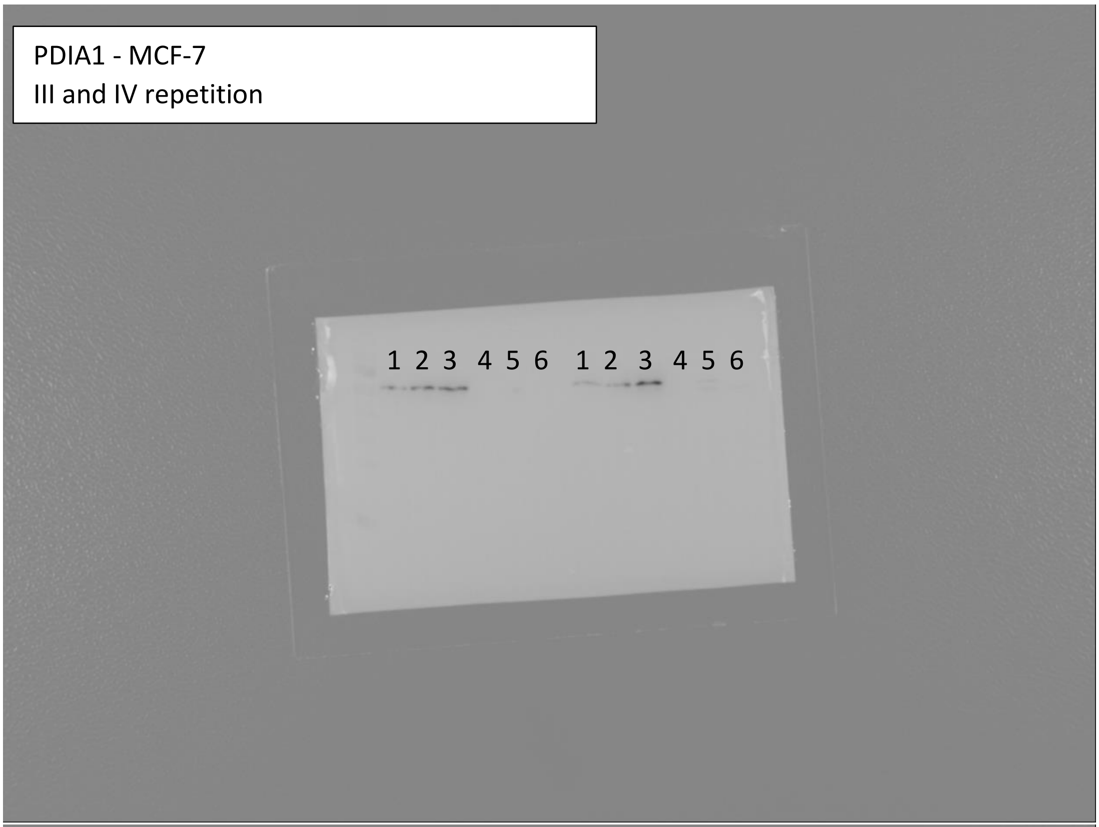

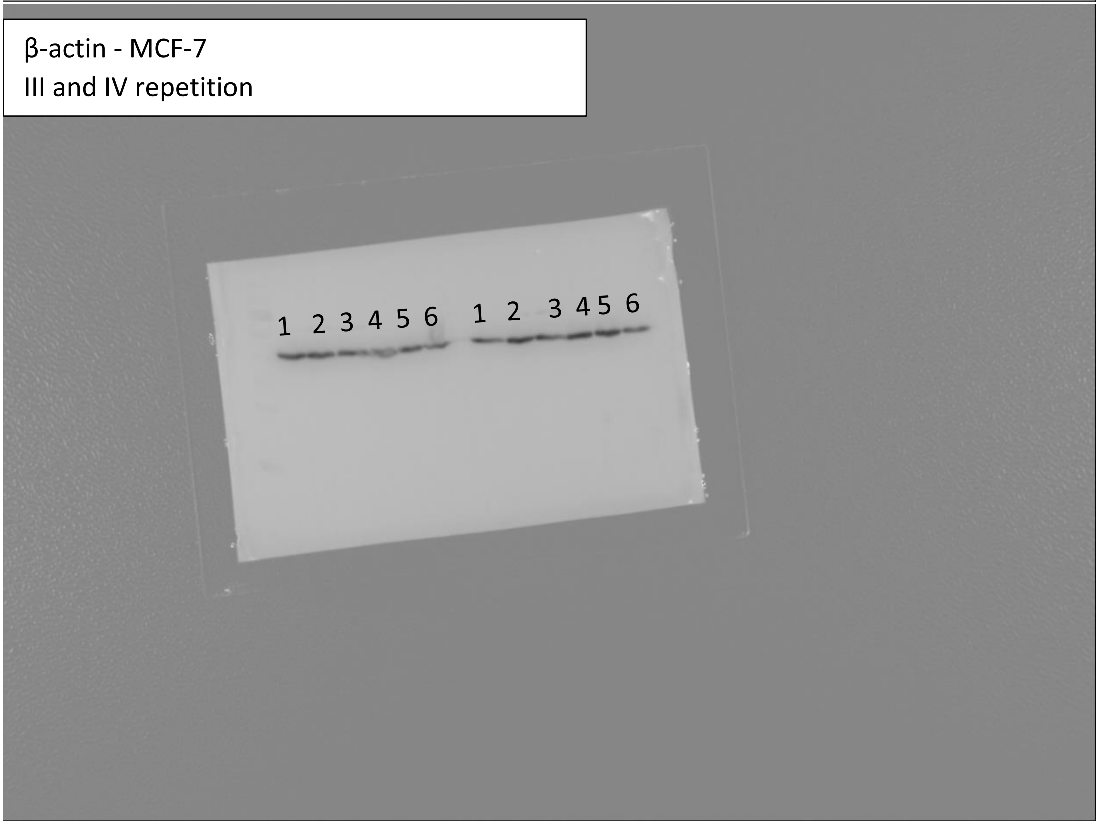

Fig. 2. PDIA1 and β-actin level in MCF-7 cells – third and fourth independent experiment.
1 – MCF-7 wt; 2 - MCF-7 shN; 3 - MCF-7 shGAPDH; 4 – MCF-7 shPDIA1-1; 5 - MCF-7 shPDIA1-2; 6 - MCF-7 shPDIA1-3.

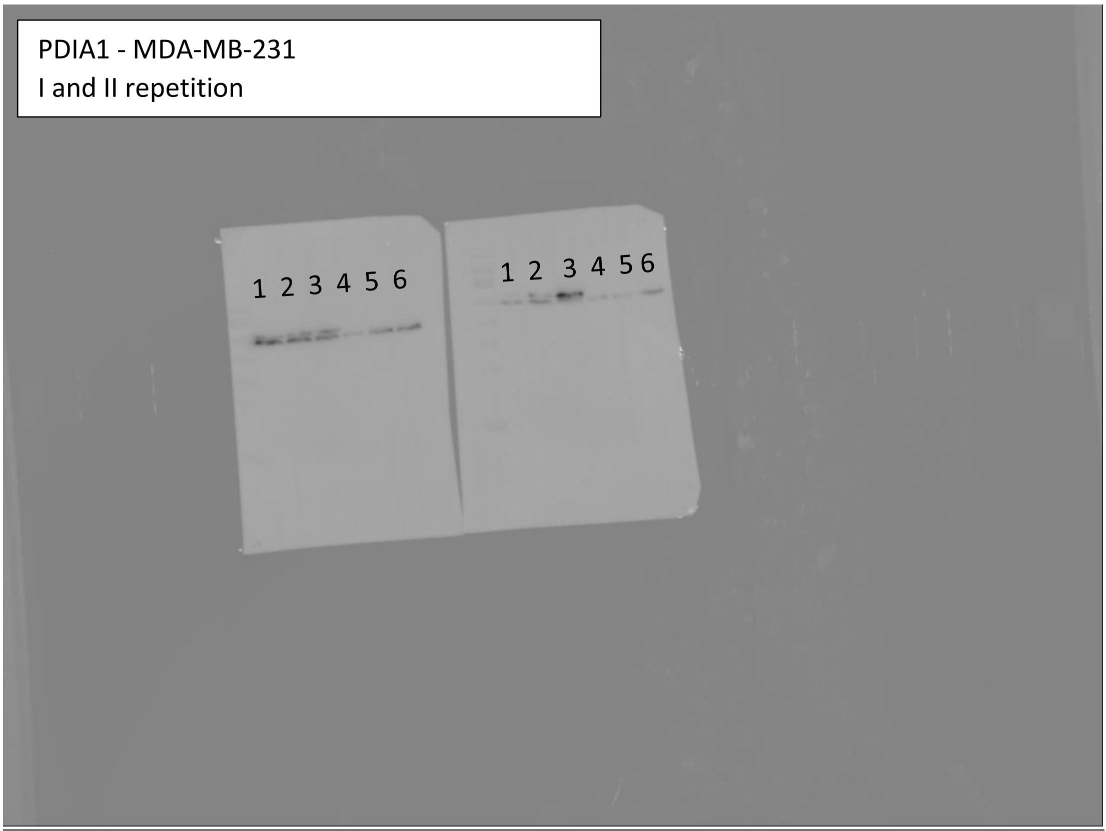

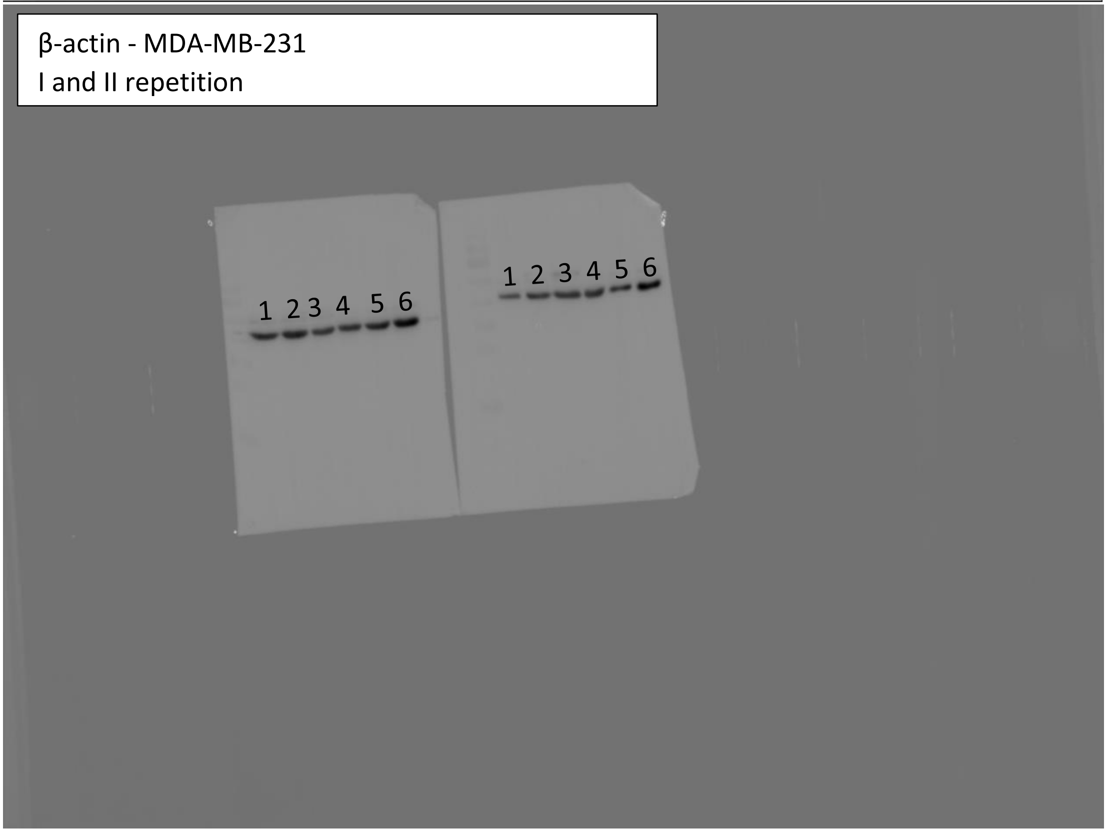

Fig. 3. PDIA1 and β-actin level in MDA-MB-231 cells – first and second independent experiment.
1 – MDA-MB-231 wt; 2 - MDA-MB-231 shN; 3 - MDA-MB-231 shGAPDH; 4 – MDA-MB-231 shPDIA1-1; 5 - MDA-MB-231 shPDIA1-2; 6 - MDA-MB-231 shPDIA1-3.

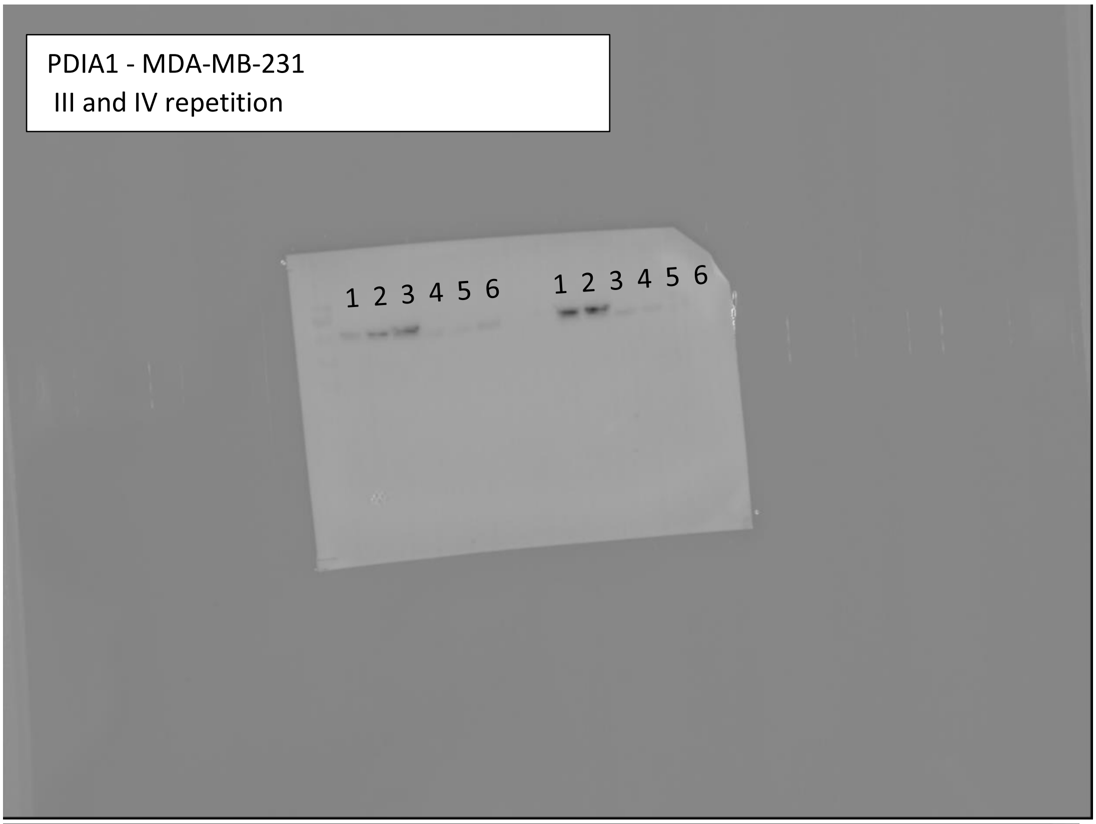

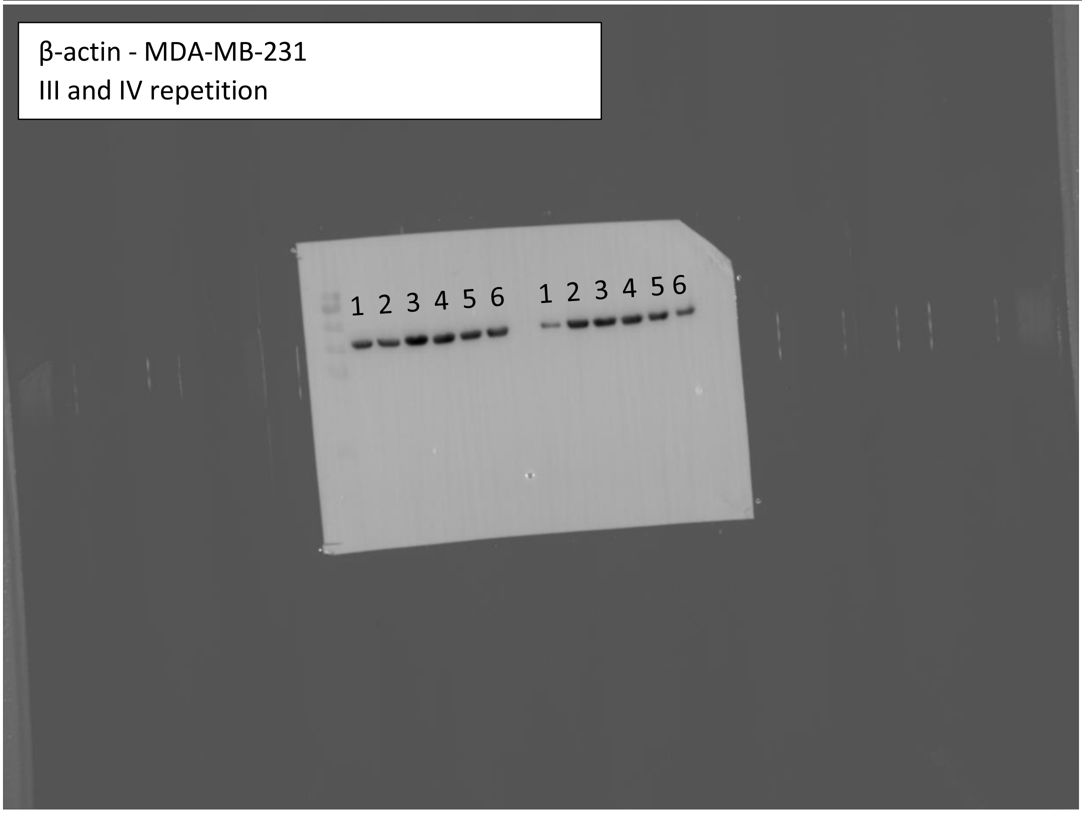

Fig. 4. PDIA1 and β-actin level in MDA-MB-231 cells – third and fourth independent experiment.
1 – MDA-MB-231 wt; 2 - MDA-MB-231 shN; 3 - MDA-MB-231 shGAPDH; 4 – MDA-MB-231 shPDIA1-1; 5 - MDA-MB-231 shPDIA1-2; 6 - MDA-MB-231 shPDIA1-3.
