# Supplementary material for: Protein Disulphide Isomerase A1 Is Involved in the Regulation of Breast Cancer Cell Adhesion and Transmigration via Lung Microvascular Endothelial Cells
Source: Cancers (Basel). 2020 Oct 2;12(10):2850. doi: 10.3390/cancers12102850 (PMC7601413; doi:10.3390/cancers12102850)
Supplement: Supplementary file 1 [file cancers-12-02850-s001.zip › Supplementary_proof/cancers-931897-supplementary-figures-proof_revised.doc]

Supplementary Figures

Protein disulphide isomerase A1 is involved in the regulation of breast cancer cell adhesion and transmigration via lung microvascular endothelial cells

Marta Stojak, Magdalena Milczarek, Anna Kurpinska, Joanna Suraj-Prazmowska, Patrycja Kaczara, Kamila Wojnar-Lason, Joanna Banach, Martyna Stachowicz-Suhs, Joanna Rossowska, Ivars Kalviņš, Joanna Wietrzyk, Stefan Chlopicki

**Figure S1.** The activity of the shPDIA1 sequence in a stable transduced breast cancer cell lines. The evaluation of PDIA1 expression at the mRNA and protein level and the proliferation rate of MCF-7 (**A**) and MDA-MB-231 (**B**) cells transduced with lentiviral vectors carrying short hairpin RNA (shRNA) against PDIA1 or negative sequence after selection with puromycin *versus* wild type cells using real-time PCR or western blot method (the expression plasmids encoded three different shRNAs sequences designed for PDIA1). The graphs represent relative density of blots (ratio of measured protein to β-actin) and the representative blots are presented below graphs. The changes in the proliferation of the cell sublines upon transduction regarding to wild type cell line were measured using SRB assay. The results are presented as the mean with SD calculated for two independent experiments for real-time PCR or at least three in the case of other methods. Statistical analysis was calculated using nonparametric Kruskal-Wallis (PDIA1 protein in MCF-7 cell sublines) followed by Dunn’s or parametric one-way ANOVA (PDIA1 protein in MDA-MB-231 cell sublines and the proliferation of MCF-7 cell sublines) Dunnett's multiple comparisons test (**p* < 0.05, ***p* < 0.01). The whole western blot figures show in figure S8.

**Figure S2.** The effect of PDIA1 inhibition by bepristat 2a on hLMVEC, MCF-7 and MDA-MB-231 cell viability assessed after 24 and 48 hours. *In vitro* MTS cytotoxicity assay showing the hLMVEC (**A, D**), MCF-7 (**B, E**) and MDA-MB-231 (**C, F**) cell viability when exposed to bepristat 2a after 24 and 48 hours of incubation. Data represent the means ± SD of at least three independent experiments. Statistical analysis was calculated using parametric one-way ANOVA followed by Dunnett's multiple comparisons test (**p* < 0.05, ***p* < 0.01, ****p* < 0.001, *****p* < 0.0001).

**Figure S3.** The effect of PDIA1 inhibition on caspase 3/7 activity in breast cancer cells. The caspase3/7 activity in MCF-7 (**A**) and MDA-MB-231 (**B**) cells treated with bepristat 2a by 24 and 48 hours as well as their activity in MCF-7 (**C**) and MDA-MB-231 (**D**) with silenced PDIA1 expression upon 24 and 48 hours after passage. Data represent the means ± SD of at least three independent experiments. Statistical analysis was calculated using nonparametric Kruskal-Wallis followed by Dunn’s multiple comparisons test (**p* < 0.05, ** *p* < 0.01, *** *p* < 0.001, **** *p* < 0.0001).

**Figure S4.** The effect of PDIA1 inhibition on cell cycle distribution of breast cancer cells. The cell cycle progression of MCF-7 (**A**) and MDA-MB-231 (**B**) cells upon 24 and 48 hours exposure to bepristat 2a. Data represent the means ± SD of three independent experiments. Statistical analysis was calculated using parametric one-way ANOVA followed by Dunnett's multiple comparisons test (**p* < 0.05, ** *p* < 0.01, *** *p* < 0.001, **** *p* < 0.0001).

**Figure S5.** The effect of PDIA1 inhibition on cell cycle distribution of breast cancer cells. The cell cycle progression of MCF-7 (**A**) and MDA-MB-231 (**B**) cells with silenced PDIA1. Data represent the means ± SD of least three independent experiments. Statistical analysis was calculated using parametric one-way ANOVA followed by Dunnett's multiple comparisons test (**p* < 0.05, ***p* < 0.01, ****p* < 0.001).

**Figure S6.** The effect of PDIA1 inhibition on ICAM-1 expression. Expression of ICAM-1 in hLMVEC cells was detected by image cytometry, as described in Supplementary Materials and Methods. Bar graph shows the percentage of positive cells in comparison with untreated control. Data are shown as means ± SD of three independent experiments carried out in six technical replicates. Statistical analysis was performed using one-way ANOVA.

**Figure S7.** The effect of PDIA1 inhibition on mitochondrial respiration. Effect of bepristat 2a on mitochondrial respiration profile (OCR) in human lung microvascular endothelial cells and breast cancer cells. Individual parameters for respiration, including basal respiration, maximal respiration, spare respiration capacity, ATP-linked oxygen consumption, proton leakage and non-mito respiration in hLMVECs (**A**), MCF-7 (**B**) and MDA-MB-231 cells (**C**). Cells were seeded in the Seahorse Bioscience microplates (20 000 cells/well for hLMVEC and MDA-MB-231; 10 000 cells/well for MCF-7). After cell seeding bepristat 2a was added to the microplates for co-incubation with cells for 24 hours, and 1 µM oligomycin, 0.8 µM FCCP and 0.5 µM/0.5 µM rotenone/antimycin A were subsequently added. Data represent the means ± SEM of two or three independent experiments (5–6 replicates in each experiment). Statistical analysis was performed using one-way ANOVA.
