# Supplementary material for: Protein Disulphide Isomerase A1 Is Involved in the Regulation of Breast Cancer Cell Adhesion and Transmigration via Lung Microvascular Endothelial Cells
Source: Cancers (Basel). 2020 Oct 2;12(10):2850. doi: 10.3390/cancers12102850 (PMC7601413; doi:10.3390/cancers12102850)
Supplement: Supplementary file 1 [file cancers-12-02850-s001.zip › Supplementary_proof/cancers-931897-supplementary-proof_revised.doc]

Supplementary Materials and Methods

Protein disulphide isomerase A1 is involved in the regulation of breast cancer cell adhesion and transmigration via lung microvascular endothelial cells

Marta Stojak, Magdalena Milczarek, Anna Kurpinska, Joanna Suraj-Prazmowska, Patrycja Kaczara, Kamila Wojnar-Lason, Joanna Banach, Martyna Stachowicz-Suhs, Joanna Rossowska, Ivars Kalviņš, Joanna Wietrzyk, Stefan Chlopicki

*Proteomic studies*

Sample preparation for proteomic studies

Cells were lysed in lysis buffer (2.8 µL/mg sample, 4 °C) containing 7 M urea (BioShop, Burlington, Ontario, Canada), 2 M thiourea (BioShop, Burlington, Ontario, Canada), mass spectrometry safe protease and phosphatase inhibitor cocktail (PIC, 1:100; Sigma-Aldrich, Steinheim, Germany) and 30 mM Tris-HCl (BioShop, Burlington, Ontario, Canada), pH 8.0, sonicated on ice and centrifuged (16,000 g, 15 minutes). The cell lysate was prepared for proteomic analysis according to Sitek et al. [1] with slight modifications. The protein concentration in the cell supernatant was assessed using Bradford assay (Bio-Rad, Hercules, CA, USA). Next, 10 µg of protein was dissolved in 258 µL of 50 mM ammonium bicarbonate (ABC; Sigma-Aldrich, Steinheim, Germany). A reduction and alkylation were performed with 45 mM dl-dithiothreitol (DTT; BioShop, Burlington, Ontario, Canada) in 50 mM ABC (15 minutes, 50 °C) and 100 mM iodoacetamide (IAA; Sigma-Aldrich, Steinheim, Germany) in 50 mM ABC (15 minutes, RT), respectively. Sequencing-grade modified trypsin (Promega, Madison, WI, USA) in a 1:50 w/w ratio was used for digestion of proteins (16 hours, 37 °C). The digestion was quenched by adding 10 µL formic acid (FA; Merck KGaA, Darmstadt, Germany). The samples were centrifuged (16,000 g, 30 minutes) and the supernatant was lyophilised.

Mass spectrometry

Peptides were reconstituted in 0.1% trifluoroacetic acid (TFA; Sigma-Aldrich, Steinheim, Germany) with 2% acetonitrile (ACN; J. T. Baker, Phillipsburg, NJ, USA) and subjected to LC-MS/MS analysis (Mass Spectrometry Laboratory at the Institute of Biochemistry and Biophysics, Polish Academy of Sciences, Warsaw, Poland). A total of 10 μL of each sample was analysed using an LC-MS system composed of a UPLC chromatograph (nanoAcquity; Waters, Milford, MA, USA) directly coupled to a QExactive mass spectrometer (Thermo Scientific, Waltham, MA, USA). Peptides were trapped on C18 pre-column (180 µm × 20 mm; Waters, Milford, MA, USA) using 0.1% FA in water as a mobile phase and then transferred to a nanoAcquity BEH C18 column (75 µm × 250 mm, 1.7 µm; Waters, Milford, MA, USA) using ACN gradient (0–35% ACN in 160 minutes) in the presence of 0.1% FA at a flow rate of 250 nL/minute. Data acquisition was performed using a data-dependent method with the top 12 precursors selected for MS2 analysis with HCD fragmentation. Dynamic exclusion was set to 30 seconds.

Identification of proteins and analysis of the proteomic data

The acquired MS/MS data were pre-processed with Mascot Distiller software (v. 2.6.2; MatrixScience, London, UK), and a search was performed with the Mascot search engine (MatrixScience, London, UK, Mascot Server 2.5) against the human proteins deposited in the Swissprot 2019_01 database (20,488 sequences). To reduce mass errors, the peptide and fragment mass tolerance settings were established separately for individual LC-MS/MS runs after a measured mass recalibration, as described previously [2]. The rest of the search parameters were as follows: enzyme, trypsin; missed cleavages, 1; fixed modifications, carbamidomethyl (C); variable modifications, oxidation (M); instrument, HCD. A statistical assessment of the confidence of peptide assignments was based on the target/decoy database search strategy, and the Mascot score threshold was adjusted to 1% false-positive results.

For the detection and semi quantitation of PDI isoforms and integrin isoforms, the exponentially modified protein abundance index (emPAI) calculation was performed [3,4] using the inbuilt tool of the Mascot search engine (MatrixScience, London, UK, Mascot Server 2.5).

*Real-Time PCR*

MDA-MB-231 and MCF-7 cell lines and their sublines (shN, shPDIA1-1, shPDIA1-2 and shPDIA1-3) were seeded at a density of 0.25×10^6^ cells/mL in 4 mL of appropriate culture medium on a 100 x 20 mm cell culture dish (Sarstedt, Nümbrecht, Germany). After 24 hours, the cells were washed twice in PBS (HIIET, Wroclaw, Poland) and collected in TRI reagent (Sigma-Aldrich, Steinheim, Germany). Next, total RNA was extracted as recommended by the manufacturer. The concentration of isolated RNA was measured using NanoDrop 2000 (Thermo Fisher Scientific, Waltham, MA, USA). Then, incubation with DNase I Amplification Grade (Invitrogen by Thermo Fisher Scientific, Waltham, MA, USA) and RNase inhibitor (EURx, Gdansk, Poland) was performed using 1 μg RNA of each sample. The process was performed at RT in Veriti 9902 thermocycler (Life Technologies, Carlsbad, CA, USA) for 15 min in order to purify of RNA. iScript cDNA synthesis kit (Bio-Rad, Hercules, CA, USA) was used for synthesizing cDNA. The synthesis protocol was as follows: 25°C for 5 minutes, 42°C for 30 minutes, 85°C for 5 minutes, and cooling down to 4°C. The samples were stored for further analysis at −20°C. The expression of P4HB (Hs01050257_m1) was analyzed using real-time PCR method. For this purpose, the TaqMan chemistry and Master Mix (all Life Technologies, Carlsbad, CA, USA) in Viia 7 (Applied Biosystems, Waltham, MA, USA) were used. The protocol was as follows: 50°C for 2 minutes, 95°C for 10 minutes; 40 cycles: 95°C for 15 seconds, 60°C for 1 minute. A total of 25 ng of cDNA was used for a single reaction and each sample was performed in triplicate in a single experiment. The experiment was conducted twice. The ΔΔCT method was used to calculate the relative changes in gene expression. The results were analyzed in ViiA 7 software Quant Studio Real-Time PCR (Life Technologies, Carlsbad, CA, USA). The expression of studied mRNA was normalized to the average expression of the two endogenous controls: RPLPO (Hs99999902_m1) and HPRT (Hs99999909_m1).

*Western blot*

MDA-MB-231 and MCF-7 cell lines and their sublines (shN, shPDIA1-1, shPDIA1-2 and shPDIA1-3) were seeded at a density of 3×10^6^ cells/mL in 6 mL of appropriate culture medium on a 100 x 20 mm cell culture dish (Sarstedt, Nümbrecht, Germany). After 24 hours, the cells were washed twice in PBS (HIIET, Wroclaw, Poland) and lysed in RIPA buffer supplemented with protease inhibitor cocktail (both from Sigma-Aldrich, Steinheim, Germany). Cell lysates were purified by centrifugation for 10 min at 4°C and 10,000 x g. Total protein concentration in cell lysates was analyzed using the modified Lowry method (Bio-Rad, Warsaw, Poland) according to the manufacturer’s protocol. Samples containing 50 μg of proteins were denatured in 4X Laemmli Sample Buffer (Bio-Rad, Warsaw, Poland) supplemented with β-mercaptoethanol (Sigma-Aldrich, Steinheim, Germany) for 5 minutes at 95°C and then separated in 12% sodium dodecyl sulphate-polyacrylamide (SDS-PAGE) gels. After electrophoresis, the proteins were transferred from gels to polyvinylidene difluoride (PVDF) membranes (0.45 μm; Merck Millipore, Burlington, MA, USA). After blocking in 5% non-fat dry milk in 0.1% TBS/Tween-20 (TBST) (TBS from HIIET, Wroclaw, Poland; Tween-20 from Sigma-Aldrich, Steinheim, Germany) at RT for 1 hour, the membranes were washed thrice for 10 minutes in 0.1% TBST and then incubated overnight at 4°C with the following primary rabbit monoclonal antibodies: anti-P4HB for PDIA1 (Abcam, Cambridge, UK). After incubation with the primary antibody, the membranes were washed four times for 5 minutes in 0.1% TBST and then incubated at RT for 1 hour with horseradish peroxidase (HRP) conjugated secondary anti-rabbit antibody (Santa Cruz Biotechnology Inc., Santa Cruz, CA, USA). After incubation, the membranes were washed four times for 5 minutes in 0.1% TBST and protein bands were detected by ECL method. Next, to determine the expression of β-actin, the same membranes were washed four times for 5 minutes in 0.1% TBST and then incubated at RT for 1 hour with 5% non-fat dry milk in 0.1% TBST. Next, the membranes were washed four times for 5 minutes in 0.1% TBST and incubated with mouse anti‑β‑actin-HRP monoclonal antibody (Santa Cruz Biotechnology Inc., Santa Cruz, CA, USA) for 1 hour at room temperature, washed four times for 5 minutes in 0.1% TBST and detected by ECL system. The results were normalized to β-actin that was used as loading control. Densitometric analysis was performed in ImageJ 1.48v software (National Institutes of Health, Bethesda, MA, USA).

*SRB assay*

The proliferation rate of the cells with silenced expression of PDIA1 was measured using sulforhodamine B (SRB) assay. MDA-MB-231 cell line and its sublines (shN, shPDIA1-1, shPDIA1-2 and shPDIA1-3) were seeded on 96-well plates (Sarstedt, Warsaw, Poland) at a density of 1×10^4^ cells/well. While MCF-7 cell line and its sublines (shN, shPDIA1-1, shPDIA1-2 and shPDIA1-3) were seeded at a density of 0.75×10^4^ cells/well. Cells were incubated in the appropriate culture medium for 72 hours. After incubation, SRB assay was performed as described by Skehan et al. [5] with minor modifications. The cells were fixed in situ by adding 50 µL per well of cold 50% trichloroacetic acid (Avantor Performance Materials, Gliwice, Poland) and were incubated at 4 °C for one hour. Following this, wells were washed five times with water and air dried. Next, 50 µL of 0.4% solution of sulforodamine B (Sigma-Aldrich, Steinheim, Germany) in 1% acetic acid (Avantor Performance Materials, Gliwice, Poland) were added to each well and plates were incubated at room temperature for 0.5 h. After incubation time, unbound dye was removed by washing plates five times with 1% acetic acid, whereas stain bound to cells was solubilized with 10 mM Tris base (Sigma-Aldrich, Steinheim, Germany). Based on raw absorbance readings at 540 nm, using Biotek Synergy H4 Hybrid Reader (Biotek Instruments, Bad Friedrichshall, Germany), mean cell proliferation inhibition were calculated as previously described by Nevozhay [6]. Compounds at each concentration were tested in triplicate in a single experiment and each experiment was repeated at least three times independently.

*MTS assay*

The effect of bepristat 2a (Sigma-Aldrich, Steinheim, Germany) on hLMVEC, MCF-7 and MDA-MB-231 cell viability was determined by the MTS tetrazolium substrate (CellTiter 96 AQueous Non-Radioactive Cell Proliferation Assay, Promega, Madison, WI, USA) according to the manufacturer's protocol. The absorbance was measured at 490 nm using a spectrophotometer (Synergy 4, BioTek, Winooski, VT, USA). Experiments were performed three times in eight technical replicates. Cellular metabolic activity changes were assessed after the hLMVEC, MCF-7 and MDA-MB-231 cells were incubated for 24 and 48 hours with tested compound.

*Caspase-3/7 activity*

MDA-MB-231 and MCF-7 cell lines and their sublines (shN, shPDIA1-1 and shPDIA1-3) were seeded on 24-well plates (Corning, Kennebunk, ME, USA) at a density of 1×10^5^ cells/well in appropriate culture medium. The test was conducted by 24 or 48 hours upon passage. Additionally, MDA-MB-231 and MCF-7 wild-type cell lines 24 hours after seeding on 24-well plates were exposed to bepristat 2a by 24 and 48 hours. The caspase-3/7 activity assay was performed as previously described by Psurski et al. [7]. After that, cells were lysed with ice-cold lysis buffer (50 mM HEPES, 10% (w/v) sucrose, 150 mM NaCl, 2 mM EDTA, 1% (v/v) Triton X-100, pH 7.3). Before applying samples to an opaque 96-well plate (Corning, Kennebunk, ME, USA), the plate was heated and filled with 160 μL of reaction buffer at the temperature of 37°C (10 μM Ac-DEVD-ACC caspase-3/7 fluorogenic substrate, 20 mM HEPES, 10% (w/v) sucrose, 100 mM NaCl, 1 mM EDTA, 10 mM DTT, 0.02% (v/v) Trition X-100, pH 7.3). Then, samples were transferred to the plate at the amount of 40 µL each. Next, sample fluorescence was continuously recorded at 37 °C for 2 h using a Biotek Synergy H4 Hybrid Reader (λ_ex_ = 360 nm, λ_em_ = 460 nm, Biotek Instruments, Bad Friedrichshall, Germany). Each experiment was repeated three times independently. Results were normalized to the protein content using the SRB method and reported as mean relative caspase-3/7 activity compared to wild-type cells or untreated cells ± SD.

*Cell Cycle analysis*

The cell cycle progression of MDA-MB-231 and MCF-7 cells and their sublines (shN, shPDIA1-1 and shPDIA1-3) was evaluated upon 24 and 48 hours incubation in the culture medium. Both cancer cell lines and their sublines (shN, shPDIA1-1 and shPDIA1-3) were seeded on 24-well plates (Corning,Kennebunk, ME, USA) at a density of 1×10^5^ cells/well in appropriate culture medium. Additionally, MDA-MB-231 and MCF-7 wild type cell lines 24 hours after seeding were exposed to bepristat 2a for 24 and 48 hours.

After incubation, the cells were collected with the use of trypsin/EDTA, pH 8 (HIIET, Wroclaw, Poland), washed and counted. Cells (1×10^6^ per sample) were then washed twice in PBS and fixed for 24 hours in cold 70% ethanol at −20°C. The cells were then washed twice in PBS and incubated with RNAse (8 μg/mL, Fermentas GmbH, St. Leon-Rot, Germany) at 37°C for 1 hour. The cells were stained for 30 minutes with propidium iodide (0.5 mg/mL; Sigma-Aldrich, Steinheim, Germany) at 4°C and the cellular DNA content was determined using BD LSR Fortessa instrument (Becton Dickinson, San Jose, CA, USA) and analyzed in a BD FACS Diva 6.2 program. The experiment was repeated at least three times.

*Expression of ICAM-1*

hLMVECs were seeded into 96-well plates (3×10^4^ cells/well), in 100 µL of medium per well and left to grow to confluence for 48-72 hours, and then incubated with hIL-1β (10 ng/mL) for 6 hours. After stimulation, endothelial cells were treated with bepristat 2a at various concentrations for 24 hours (1, 5, 10, 50 µM). Then, hLMVECs were incubated in the dark with mouse anti-human CD54-PE (BD Pharmingen, San Jose, CA, USA) according to the manufacturer's protocol and stained with Hoechst 33,342 (Life Technologies, Carlsbad, CA, USA) for 10 minutes. The expression of surface ICAM-1 molecule was observed by CQ1 image cytometer (Yokogawa, Tokyo, Japan) in six randomly selected visual fields for each well. The images were analyzed using Columbus 2.4.2 Software (Perkin Elmer, Waltham, MA, USA). Experiments were performed three times in six technical replicates.

*Analysis of Cellular Bioenergetics by Extracellular Flux Technology*

To measure mitochondrial function and glycolysis in adherent endothelial hLMVEC and cancer MDA-MB-231 and MCF-7 cells, a Seahorse Bioscience XFe 96 Analyzer (Santa Clara, CA, USA) was used. Cells were seeded into Seahorse XFe 96-well plates approximately 24 hours before the experiment, according to the Seahorse protocol. The optimal cell density per well for all cell lines were selected in preliminary experiments. For hLMVEC and MDA-MB-231, densities of 2×10^4^ cells/well while for MCF-7 density of 1×10^4^ cells/well were chosen, respectively. After cell seeding, they were left to grow for 24 h and then cells were incubated with bepristat 2a at various concentrations (1, 5, 10, 30, 50 µM) for 24 h. One day before the experiment, sensor cartridges were hydrated in XF calibrant and maintained at 37°C in air without CO_2_. On the day of the experiment, cells were washed twice and incubated with bicarbonate-free low buffered assay medium for 1 hour at 37°C in air without CO_2_ prior to the beginning of the assay. The assay medium was freshly prepared on the day of the experiment by supplementation of XF Base Medium Minimal DMEM with metabolic substrates as in culture media used for propagation of these cell lines: glucose (1 g/L for hLMVEC, 2 g/L for MDA-MB-231 and MCF-7), glutamine (2 mM for all cell lines) and sodium pyruvate (1 mM for hLMVEC, 2 mM for MDA-MB-231 and MFC-7); pH was adjusted to 7.4 with NaOH. Changes in oxygen consumption rate (OCR) and extracellular acidification rate (ECAR) were assessed over time by sequential injections of vehicles, 1 µg/mL oligomycin in port A, 0.8 µM FCCP (Sigma-Aldrich, Steinheim, Germany) in port C and 0.5 µM/0.5 µM rotenone/antimycin (both from Sigma-Aldrich, Steinheim, Germany) A in port C. Concentrations of oligomycin, FCCP, rotenone and antymycin A were optimized separately for both cell lines in preliminary experiments. Afterwards, cells were lysed in 0.1% Triton in PBS and protein content was measured by BCA assay. This assay was performed twice with six replicates per condition. From the mitochondrial stress test, we determined key parameters of mitochondrial function by using a Seahorse XF Cell Mito Stress Test Report Generator for calculation of OCR values linked to: basal respiration, acute response, proton leak, maximal respiration, spare respiratory capacity, ATP production and non-mitochondrial respiration.

References:

1. Sitek, B.; Waldera-Lupa, D.M.; Poschmann, G.; Meyer, H.E.; Stühler, K. Application of label-free proteomics for differential analysis of lung carcinoma cell line A549. *Methods Mol. Biol.* **2012**, *893*, 241–248, doi:10.1007/978-1-61779-885-6_16.
2. Malinowska, A.; Kistowski, M.; Bakun, M.; Rubel, T.; Tkaczyk, M.; Mierzejewska, J.; Dadlez, M. Diffprot - software for non-parametric statistical analysis of differential proteomics data. *J. Proteomics* **2012**, *75*, 4062–4073, doi:10.1016/j.jprot.2012.05.030.
3. Ishihama, Y.; Oda, Y.; Tabata, T.; Sato, T.; Nagasu, T.; Rappsilber, J.; Mann, M. Exponentially modified protein abundance index (emPAI) for estimation of absolute protein amount in proteomics by the number of sequenced peptides per protein. *Mol. Cell. Proteomics* **2005**, *4*, 1265–1272, doi:10.1074/mcp.M500061-MCP200.
4. Roy, J.; Wycislo, K.L.; Pondenis, H.; Fan, T.M.; Das, A. Comparative proteomic investigation of metastatic and non-metastatic osteosarcoma cells of human and canine origin. *PLoS One* **2017**, *12*, doi:10.1371/journal.pone.0183930.
5. Skehan, P.; Storeng, R.; Scudiero, D.; Monks, A.; McMahon, J.; Vistica, D.; Warren, J.T.; Bokesch, H.; Kenney, S.; Boyd, M.R., New Colorimetric Cytotoxicity Assay for Anticancer-Drug Screening;., J. Natl. Cancer Inst., 1990, 82(13), 1107-1112, doi.org/10.1093/jnci/82.13.1107.
6. Nevozhay, D. Cheburator software for automatically calculating drug inhibitory concentrations from in vitroscreening assays. *PLoS One* **2014**, *9*, doi:10.1371/journal.pone.0106186.
7. Psurski, M.; Janczewski, Ł.; Świtalska, M.; Gajda, A.; Goszczyński, T.M.; Oleksyszyn, J.; Wietrzyk, J.; Gajda, T. Novel phosphonate analogs of sulforaphane: Synthesis, in vitro and in vivo anticancer activity. *Eur. J. Med. Chem.* **2017**, *132*, 63–80, doi:10.1016/j.ejmech.2017.03.028.
